# Alpha-lipoic acid alters the antitumor effect of bortezomib in melanoma cells in vitro

**DOI:** 10.1038/s41598-020-71138-z

**Published:** 2020-08-31

**Authors:** Angéla Takács, Eszter Lajkó, Orsolya Láng, Ildikó Istenes, László Kőhidai

**Affiliations:** 1 Department of Genetics, Cell- and Immunobiology, Semmelweis University, Budapest, Hungary; 2 1st Department of Internal Medicine, Semmelweis University, Budapest, Hungary

**Keywords:** Cancer therapy, Cell death, Cell division, Melanoma

## Abstract

Bortezomib (BOZ) is a proteasome inhibitor chemotherapeutic agent utilized to treat multiple myeloma and recently offered to cure melanoma. Bortezomib-induced neuropathy is one of the dose-limiting side-effects, which can be treated with antioxidants (e.g. alpha-lipoic acid—ALA and Vitamin B1—vit B1). We hypothesized that these antioxidants may counteract the antitumor activity by disrupting the BOZ-induced pathways (e.g. proteasome inhibition or reactive oxygen species generation). The objectives were: (i) to verify the anti-proliferative effect of BOZ; (ii) to compare the influence of the antioxidants on the antitumor effect of BOZ in melanoma (A2058) and myeloma (U266) cells. At first, the reduction in the anti-proliferative effect of BOZ by ALA was proved in melanoma cells. Analysis of p53 phosphorylation and the cell cycle progression revealed that ALA failed to counteract these effects of BOZ. Nevertheless, a good correlation was found between the inhibition of the anti-proliferative effect, the anti-proteasome activity and the oxidative stress level after the co-treatment with 20 ng/mL BOZ + 100 μg/mL ALA. Downregulation of apoptotic proteins such as HO-1 and Claspin along with the inhibition of the cleavage of Caspase-3 indicated the proteomic background of the altered responsiveness of the melanoma cells exposed to BOZ + ALA. This phenomenon draws attention to the proper application of cancer supportive care to avoid possible interactions.

## Introduction

Bortezomib (BOZ), a targeted proteasome inhibitor has become an unavoidable medicine in the treatment of multiple myeloma, a hematological malignancy. The U. S. Food and Drug Administration (FDA) approved BOZ for the treatment of progressive multiple myeloma in 2003. The second-line approval of BOZ was in 2005, so since that time BOZ is available for patients who had already minimum one unsuccessful treatment^1^. Since 2008, BOZ was put in practice for the treatment of active multiple myeloma as the first-line therapy in combination with e.g. dexamethasone and cyclophosphamide or dexamethasone and lenalidomide^2^. In addition, recently scientists have investigated the potential activity of BOZ and its probable combinations with the purpose of curing solid tumor malignancies, e.g. melanoma, the malignant transformation of melanocytes^3–5^.

BOZ is a low molecular weight, hydrophilic dipeptide-boronic acid derivative. It acts as a reversible inhibitor of the chymotrypsin-like protease activity of the proteasome^6^. It blocks the NF-κB (nuclear factor kappa-light-chain-enhancer of activated B cells) pathway via inhibition of the degradation of the IκB protein, that binds to the NF-κB and blocks its transport to the nucleus^7,8^. BOZ has several other antineoplastic functions: it arrests the cell cycle in G2/M phase, activates the pro-apoptotic endoplasmic reticulum stress—reactive oxygen species (ROS) associated cascade and upregulates some pro-apoptotic factor-like NOXA (phorbol-12-myristate-13-acetate-induced protein 1)^8–10^.

In general, chemotherapy-induced peripheral neuropathy (CIPN) is a severe side-effect of many various chemotherapeutic agents, such as BOZ, cisplatin or paclitaxel^11^. Unfortunately, bortezomib-induced peripheral neuropathy (BIPN) is a type of CIPN and belongs to the dose-limiting side effects of BOZ^12,13^. Seventy-five percent of the patients suffering from multiple myeloma had grade 1–2 BIPN and in 12% of BOZ-treated myeloma patients developed grade 3–4 BIPN^11^. The incidence of peripheral neuropathy was 50% among patients suffering from solid tumors, e.g., melanoma and treated with BOZ^14^. The symptoms of BIPN are paresthesia, allodynia, hyperalgesia^11^. These symptoms are usually presented in stocking-and glove-shaped distribution, and thus dose reduction or discontinuation can be required^12^. There have been various supposed theories about the pathogenesis of BIPN. It can be caused by the rising ROS levels, inducing tubulin polymerization, stabilizing microtubules and blocking the NF-κB pathway^13,15^. These effects can be observed in the peripheral nerves that lead to demyelination^16^. To optimize the quality of life of the patients, different types of CIPN can be treated with antiepileptic agents, such as gabapentin, antidepressants, such as amitriptyline and neuroprotective or antioxidant vitamins, such as Vitamin C^17,18^. These antioxidant vitamins are over-the-counter (OTC) products and are the part of the cancer supportive care that has a growing market nowadays. However, cancer supportive care is aimed to focus on the quality of life during the treatments of cancerous illnesses but many times it may also decrease the antitumor effect of the chemotherapeutic agent. Patients can buy these OTC products in order to help to manage the symptoms of chemotherapy-related side effects without recognizing the potential risks. Several studies have been carried out on the antagonizing effect of Vitamin C on the anticancer effect of antineoplastic medicines^17,19^.

Other neuroprotective agents, e.g. alpha-lipoic acid (ALA) and Vitamin B1 (vit B1) are also applied as advantageous agents for cancer supportive care; previous studies have shown the positive effect of these compounds mentioned above in different neurodegenerative conditions, e.g. diabetic neuropathy or anticancer drug-related peripheral neuropathy^20–22^.

Based on the aforementioned findings, we hypothesized that the antioxidants may counteract the antitumor activity of BOZ. In the present work our aim was (i) to verify the anti-proliferative effects of BOZ in melanoma and myeloma cell lines (ii) to test the influence of the ALA and vit B1 on the tumor growth inhibitory effect of BOZ as well as (iii) to study the mechanisms of the effects of BOZ + antioxidants co-treatments.

## Results

### Alpha-lipoic acid influences the anti-proliferative effect of BOZ

**Figure 1:**
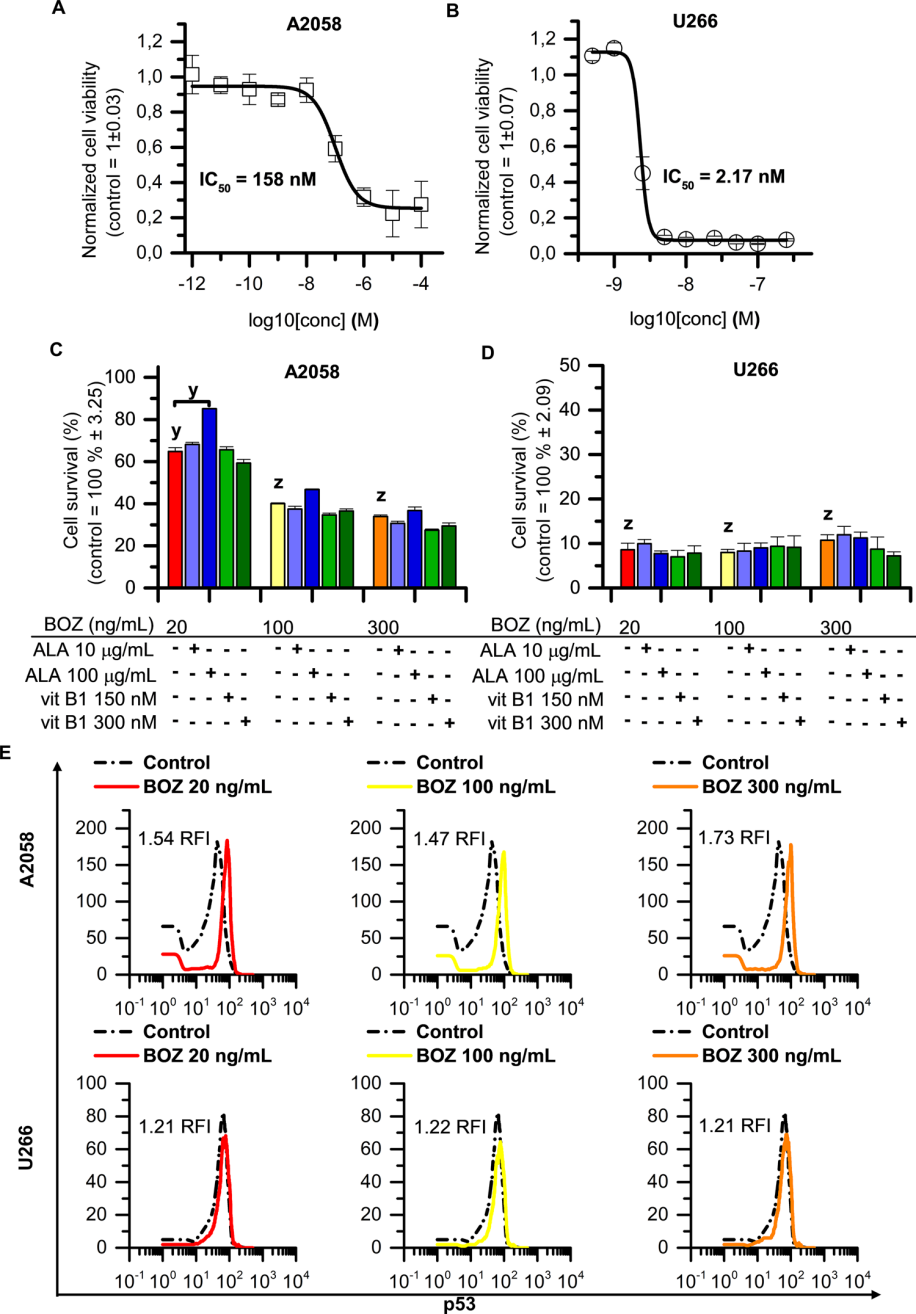
Alpha-lipoic acid counteracts the anti-proliferative effect of bortezomib on melanoma cells. Concentration–response curves for (**A**) A2058 and (**B**) U266 cells treated with BOZ for 24 h. The data are normalized to the control wells. The IC_50_ value of BOZ was determined by fitting a sigmoidal dose–response curve to the data, using Origin Pro 8.0. Data are given as mean values ± standard deviation (SD), (n = 3). Influence of alpha-lipoic acid and vitamin B1 on bortezomib mediated cell death on A2058 (**C**) and U266 cells (**D**) after 24 h incubation. Cells were treated with therapeutic agents as indicated. Data are given as mean values ± standard deviation (SD), (n = 3). (**E**) Analysis of the level of the activated p53 of A2058 and of U266 cells after 24 h incubation with 20, 100 and 300 ng/mL bortezomib (BOZ). The RFI (ratio of mean fluorescence intensity) is reported for each histogram. The figure shows representative data of 2 identical experiments. The levels of significance are shown as follows: x: P < 0.05; y: P < 0.01; z: P < 0.001, determined by the One-way ANOVA test followed by Fishers LSD post hoc test.

First, we determined the IC_50_ values in the 24th hour of the BOZ treatment on both cell lines (A2058—IC_50_: 158 nM; U266—IC_50_: 2.17 nM). Based on these IC_50_ values, the A2058 melanoma cell line showed a 72-fold less sensitivity to BOZ compared with the U266 myeloma cells (Fig. 1A,B). In the further experiments with the binary combination of BOZ and antioxidant vitamins, these compounds were tested only in representative concentrations (BOZ: 20, 100, 300 ng/mL; ALA: 10, 100 µg/mL and vit B1: 150, 300 nM). The concentrations were chosen based on their maximal plasma concentrations (C_max_) that occur after the clinical use^23–25^, since in case of BOZ, the C_max_ was indicated to determine the incidence of BIPN. Mu et al. have found that the incidence of BIPN was decreased compared to the intravenous administration of the same dose and the underlying mechanism might be associated with lower C_max_ in case of the subcutaneous administration^26^.

Figure 1C,D shows the BOZ-induced anti-proliferation on myeloma and melanoma cells following 24 h exposure with 20, 100 and 300 ng/mL BOZ. The BOZ was anti-proliferative on the melanoma cell line A2058 in a dose-dependent manner (Fig. 1C), while on the myeloma cell line U266, in all tested concentrations with the same extent (Fig. 1D). It is clearly shown in Fig. 1C that the number of viable melanoma cells was significantly increased following 20 ng/mL BOZ + 100 µg/mL ALA co-treatment compared to the only-BOZ treated cells, however, the antioxidants alone were found to have minimal impact on the proliferation of the melanoma cells (Supplementary Fig. S1A). The IC_50_ value of BOZ on A2058 cells (160 nM) and the combined effect of 20 ng/mL BOZ + 100 µg/mL ALA were validated using CompuSyn Combined Index equation algorithms (Supplementary Figure S2) and an antagonism was detected (Combination Index = 8.67) (Supplementary Fig. S2B).

In case of U266 cells the antioxidants could not influence the antitumor effect of any BOZ treatment (Fig. 1D), but alone ALA 10 μg/mL was able to inhibit the proliferation of the cells (Supplementary Fig. S1B). As shown in Fig. 1E dose-escalated BOZ treatment induced remarkable p53-activation (phosphorylation of p53) even at lower concentrations on melanoma cell line compared to the control group (RFI = BOZ treated cells p53 MFI/control cells p53 MFI; RFI: ratio of the mean fluorescence intensity; MFI: mean fluorescence intensity). However, BOZ did not stimulate p53-activation in U266 cells. From the data in Supplementary Table S1, it is apparent that the antioxidants could barely change the status of the p53 in the cells treated with them as monotherapy as well as when they were in co-treatments with the different concentrations of BOZ.

**Figure 2:**
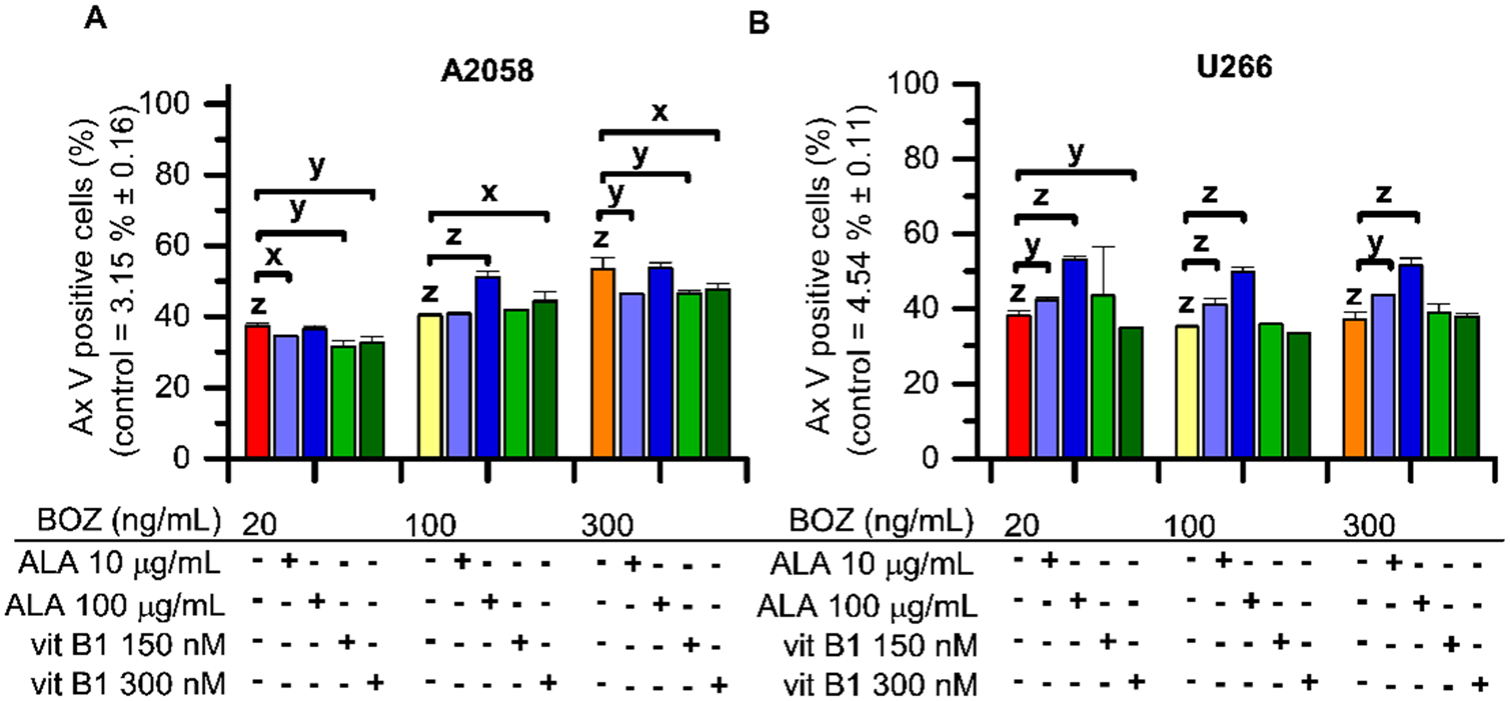
Bortezomib (BOZ) induced the percentage of early apoptotic (Annexin V positive) cells, that effect was slightly altered by the co-treatments with alpha-lipoic acid (ALA) or vitamin B1 (vit B1). Apoptosis in A2058 (**A**) and in U266 (**B**) cells after 24 h long incubation with 20, 100 and 300 ng/mL bortezomib and combinations of BOZ + 10 or 100 μg/mL ALA and 150 or 300 nM vit B1 analyzed by Annexin V assay. Data are given as mean values ± standard deviation (SD) (n = 2). The levels of significance are shown as follows: x: P < 0.05; y: P < 0.01; z: P < 0.001, determined by the One-way ANOVA test followed by Fishers LSD post hoc test.

As can be seen in Fig. 2, the percentage of the Annexin V (Ax V) positive cells was increased by every BOZ treatment in both cell lines. Surprisingly, the antioxidants were able to further increase the percentage of the early apoptotic cells compared to the matching BOZ-treated control in many instances, e.g., A2058 cell: 100 ng/mL BOZ + 100 μg/mL ALA or 150 nM vit B1; U266 cell: 20, 100, 300 ng/mL BOZ + 10 or 100 μg/mL ALA, respectively. Both concentrations of vit B1 could reduce the percentage of Ax V postive A2058 cells, however, no significant decrease was observed in the 20 ng/mL BOZ + 100 μg/mL ALA combination compared to the 20 ng/mL BOZ-treated cells (Fig. 2.A). The mono treatments with 100 μg/mL ALA or 300 nM vit B1 resulted in the induction of apoptosis (Supplementary Fig. S3A, B).

**Figure 3:**
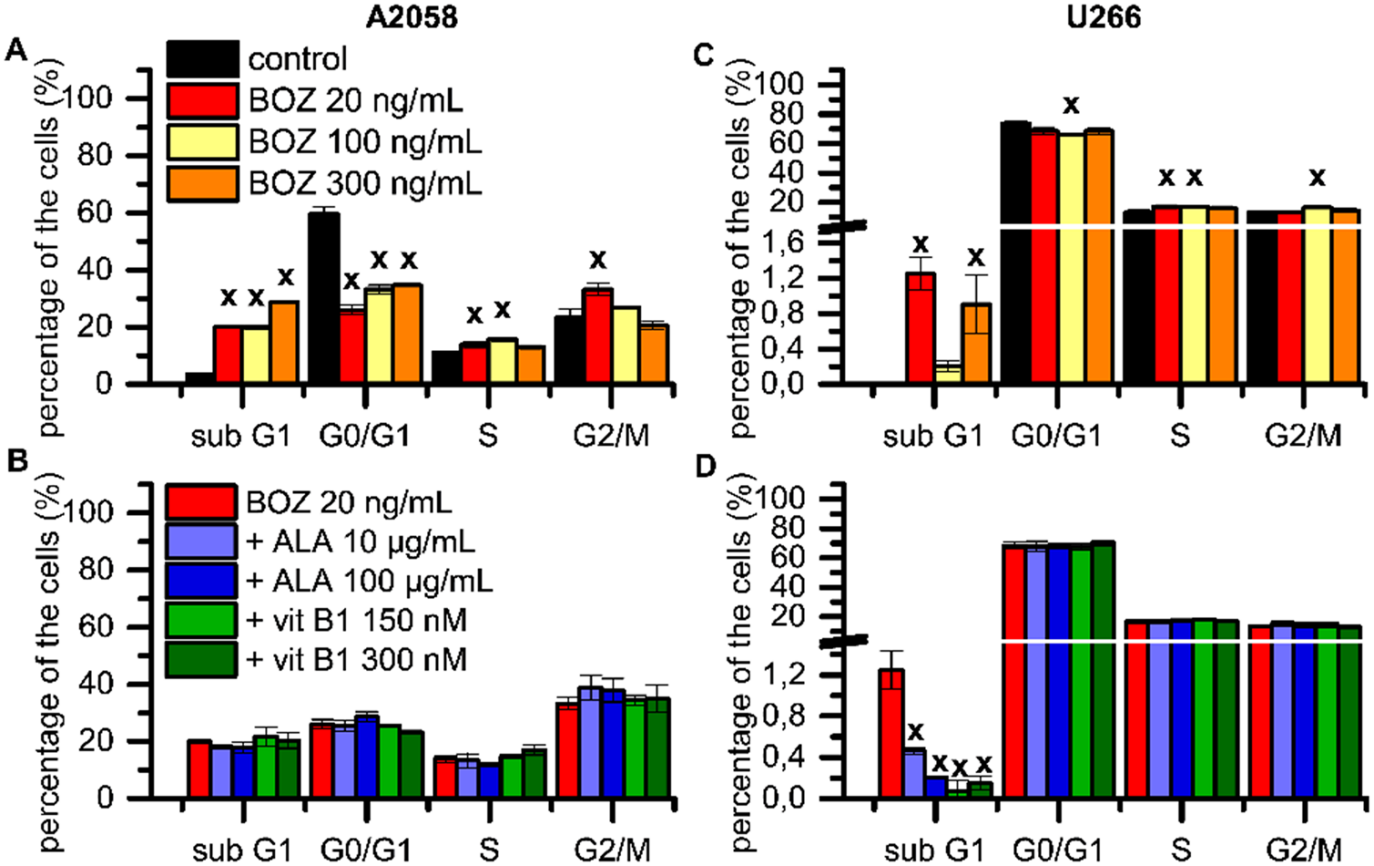
Alpha-lipoic acid failed to inhibit the progression of the cell cycle induced by bortezomib treatment. Cell cycle analysis of A2058 (**A-B**) and U266 (**C-D**) cell lines after 24 h long incubation with 20, 100 and 300 ng/mL bortezomib (BOZ) and combinations of 20 ng/mL BOZ + 10 or 100 μg/mL alpha-lipoic acid (ALA) and 150 or 300 nM vitamin B1 (vit B1) analyzed by NucleoCounter. Data are given as mean values ± standard deviation (SD) (n = 2). The levels of significance are shown as follows: x: P < 0.05; y: P < 0.01; z: P < 0.001, determined by the One-way ANOVA test followed by Fishers LSD post hoc test.

We then hypothesized that BOZ may affect the cell cycle of the myeloma and melanoma cells. The cell cycle profile was evaluated after 24 h in the presence of BOZ (Fig. 3). The percentage of A2058 cells in the sub G1 phase increased following the treatment with the all BOZ concentrations, along with a decrease in the proportion of the resting cells from G0/G1 phase (Fig. 3A). The treatment with 20, 100 and 300 ng/mL BOZ resulted in a tendentious increase in the proportion of cells in the S phase and G2/M phase. The cell cycle profile of A2058 cells was not influenced as the result of antioxidant mono treatments (Supplementary Fig. S5A). In contrast to our cell viability results, ALA could not significantly inhibit the effect of 20 ng/mL BOZ on the sub G1 phase (Fig. 3B). The effect of the antioxidant co-treatments was insignificant on the G2/M arrest induced by 20 ng/mL BOZ treatment.

In the case of U266 myeloma cells, the test revealed, that the number of cells in the sub G1 phase was slightly but significantly increased by the exposure to 20 and 300 ng/mL BOZ (Fig. 3C). This apparent lack of correlation between the results of the cell cycle and the cell viability can be attributed to the robust changes of the cell morphology during apoptosis. As the apoptotic bodies can also contain DNA fragments, the loss of these morphologically changed particles during the sample preparation may complicate the separation of the phases^27,28^. BOZ 20 and 100 ng/mL seemed to arrest the cell cycle in the S-phase and due to the 100 ng/mL BOZ treatment a slight, but significant increase in the proportion of cells in G2/M phase was also observed (Fig. 3C). This subG1 phase increasing effect of 20 ng/mL BOZ was significantly inhibited by all antioxidant co-treatments (Fig. 3D). The rest of the combinations resulted in a similar cell cycle progression as it was detected in the case of the cells treated with BOZ alone. Nevertheless, there are two exceptions where vit B1 antagonized the effect of 100 and 300 ng/mL BOZ (Supplementary Fig. S4C, D), while the mono treatment of myeloma cells with the antioxidants failed to cause any drastic cell cycle changes (Supplementary Fig. S5B).

### Alpha-lipoic acid could partly antagonize the inhibition of chymotrypsin-like proteasome activity of BOZ in melanoma cells

**Figure 4:**
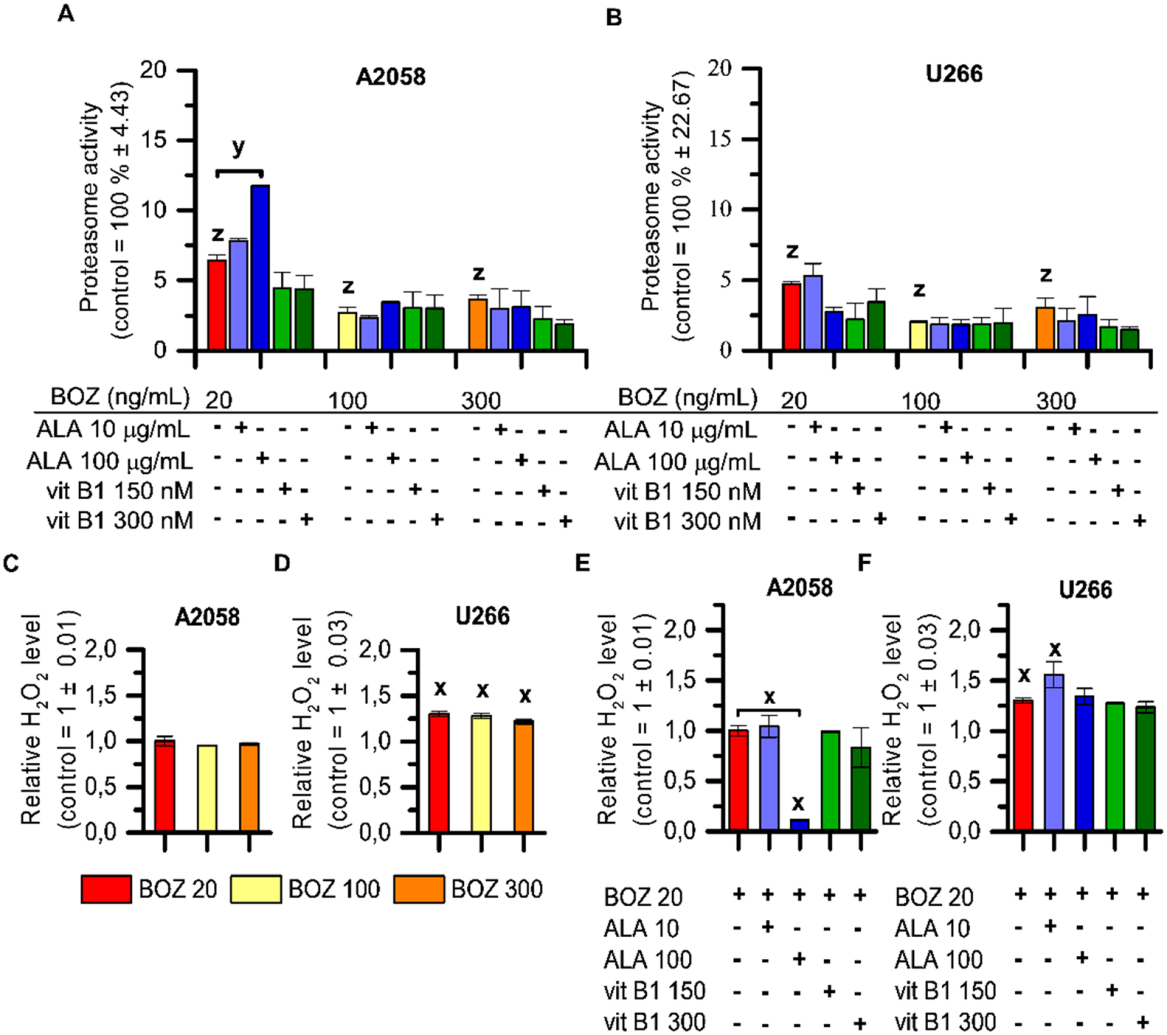
Increasement in the proteasome activity following co-treatment with ALA + BOZ is concentration-dependent and correlates with the results on the cell viability. The effect of bortezomib (BOZ) and its combinations with antioxidants on the chymotrypsin-like activity of the proteasome in A2058 (**A**) and in U266 (**B**) cells. Cells were treated with therapeutic agents as indicated for 24 h. The data were normalized to the control wells. Data are given as mean values ± standard deviation (SD), (n = 3). A2058 (**C,E**) or U266 cells (**D,F**) were treated as indicated and then the relative H_2_O_2_ level of the cells was determined. The data are normalized to the control wells. Mean ± SD (n = 2). The levels of significance are shown as follows: x: P < 0.05; y: P < 0.01; z: P < 0.001, determined by the One-way ANOVA test followed by Fishers LSD post hoc test.

Before analyzing the effect of the BOZ and antioxidant combinations on the anti-chymotrypsin-like proteasome activity of BOZ, we determined the IC_50_ values of the BOZ following a 24 h incubation in both cell lines (Supplementary Fig. S8A, B). A2058 cells (IC_50_ = 4.39 nM, Supplementary Fig. S8A) tend to have a slightly lower sensitivity to BOZ than U266 cells (IC_50_ = 1.49 nM, Supplementary Fig. S8B). In both A2058 and U266 cells, the investigated concentrations (20, 100, 300 ng/mL) of BOZ had a great inhibition on the proteasome activity, but concentration dependence could not be observed (Fig. 4A,B). The proteasome activity of the cells was not significantly altered after the treatment with any of the antioxidants as mono treatment (Supplementary Fig. S8C, D). The antioxidants had barely any antagonizing effect on the proteasome inhibition of BOZ, except for one combination, where enlargement in the percentage of the chymotrypsin-like protease activity of the proteasome could be monitored following 100 μg/mL ALA + 20 ng/mL BOZ co-treatment in the melanoma cells (Fig. 4A).

**Figure 5:**
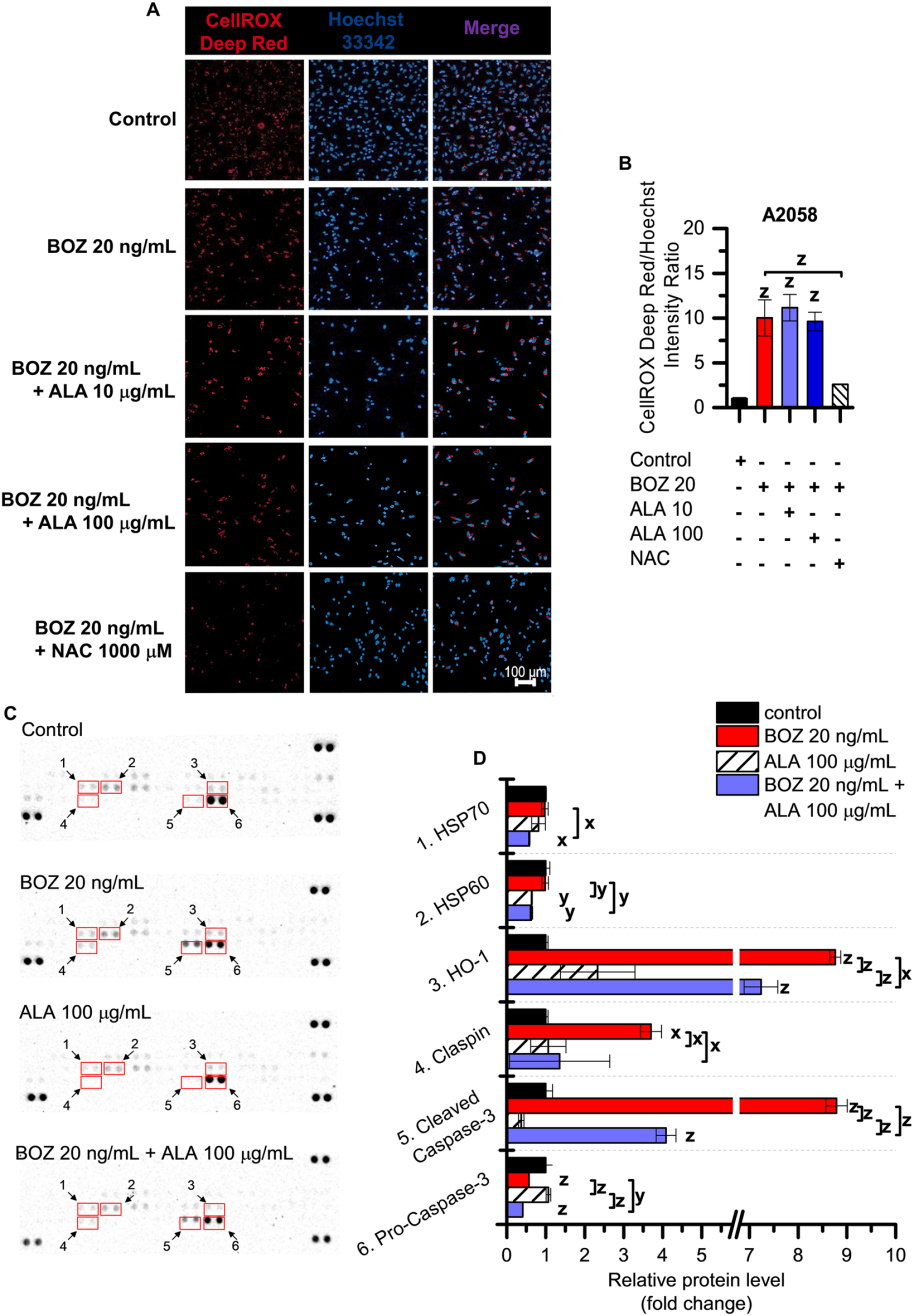
Bortezomib induces oxidative stress in melanoma cells and inhibits cleavage of Caspase-3. (**A**) Melanoma cells were treated as indicated for 24 h. The cells were imaged on Zeiss Celldiscoverer 7 using 10 × magnification. The scale bar represents 100 μm. (**B**) Comparison of the intensity values of the red channel normalized to the cell nuclei. The data were analyzed using ImageJ software. Data in duplicates were expressed as mean ± standard deviation. (**C**) Proteome profiling of A2058 cells after treated with 20 ng/mL BOZ, 100 μg/mL ALA and their combination for 24 h. On the left panel, the array blots are presented. Spots for each protein are in duplicate. Red squares indicate the proteins quantified on the right panel. (**D**) Relative protein levels were analyzed using Image Lab 6.0.1 Software. The levels of significance are shown as follows: x: P < 0.05; y: P < 0.01; z: P < 0.001, determined by the One-way ANOVA test followed by Fishers LSD post hoc test.

According to the luminescent based assay, 20, 100 and 300 ng/mL BOZ can induce H_2_O_2_ generation in the myeloma cells in the twenty-fourth hour of the BOZ treatment, but not in the melanoma cells (Fig. 4C,D). The antioxidants could not affect the H_2_O_2_ inducer activity of BOZ in U266 cell line, while in the A2058 cells 100 μg/mL ALA was able to reduce the basal quantity of H_2_O_2_ compared to the control or to the cells treated with 20 ng/mL BOZ (Fig. 4E,F). It could be proved also by automated high-throughput microscopy (Zeiss Celldiscoverer 7), that A2058 cells normally have a high ROS content located in the cytoplasm that can be prevented by the treatment with negative control, the ROS scavenger N-Acetyl-L-cysteine (NAC) (Fig. 5A,B). Based on the microscopic results, 20, 100 and 300 ng/mL BOZ increased the intensity of the red channel (Supplementary Fig. S9A, B). To validate the analysis, a positive control, 150 μM tBHP was added to a set of the cells and caused a significant increase in the intensity ratio compared to the medium control and this increase was reduced by the negative control, 1,000 μM NAC (Supplementary Fig. S9A, B). To confirm the ROS-generation effect of BOZ, A2058 cells were treated with the combination of 20 ng/mL BOZ + 1,000 μM NAC (Fig. 5A). This resulted in an intensity decrease of the red channel compared to the only-BOZ treated cells (Fig. 5B) proving the ROS generation effect of BOZ. Contrary to expectations, ALA was not able to decrease the intensity of the red channel in combination with BOZ (Fig. 5A,B), but interestingly lower intensity values were observed in cases with vit B1 (Supplementary Fig. S10). The basal oxidative activity of the A2058 cells was not significantly altered in cases of ALA or vit B1 mono treatments (Supplementary Fig. S11).

Proteomic analysis was done to identify the changes in the relative level of the apoptosis-related proteins by 20 ng/mL BOZ, 100 μg/mL ALA and their combination compared to the control. According to Fig. 5C, after the exposure to the BOZ + ALA combination, there were 6 identified proteins compared to the BOZ-treated cells (HSP70, HSP60, HO-1, Claspin, cleaved Caspase-3, pro-Caspase-3), where the intensity of the protein spots was down-regulated. The intensities of the protein spots were quantified and the relative protein levels (fold change) compared to the same protein spots found on the control membrane were determined and presented in Fig. 5D. Among these proteins, we observed significantly increased amount of HO-1, Claspin and cleaved-Caspase-3 in BOZ-treated A2058 cells. However, the expression of HSP70 and HSP60 was not altered by the BOZ treatment and the expression of pro-Caspase-3 was down-regulated by BOZ compared to the untreated cells. The Supplementary Figure S12 details the data on the relative level of these proteins in U266 cells. This figure highlights, that the expression of HO-1 and cleaved-Caspase-3, along with the expression of HSP60 and HSP70, is higher compared to the medium control. Unlike the A2058 cells, Claspin was not altered by 20 ng/mL BOZ, and co-treatment of 20 ng/mL BOZ + 100 μg/mL ALA could downregulate the HSP60 and HSP70 level, but it did not alter the HO-1 and the cleaved-Caspase-3 level.

## Discussion

Bortezomib, the first FDA approved proteasome inhibitor drug, is very important for the first-line therapy of multiple myeloma^2^. There is a large volume of studies attempting to explain the effects of BOZ on U266 myeloma cells^29–31^. As Chen pointed out, BOZ increased Ax V positivity and inhibited the growth of the U266 cells. In 2013, Selimovic et al. published a paper in which they described BOZ induced apoptosis- and autophagy-related pathways in melanoma cells, too^32^.

According to Larsson et al.^33^, the IC_50_ values of drugs are suitable drug response metrics to predict the sensitivity of cells. In the present work, we report that U266 myeloma cells were (IC_50_: 2.17 nM) more sensitive to BOZ than A2058 melanoma cells (IC_50_: 158 nM). Our data demonstrate a tumor-specific, antitumor effect of BOZ, because p53 activation was observed only on melanoma but not on myeloma cell lines. These results are consistent with other studies and suggest that, depending on the tumor type, BOZ may act independently of p53 phosphorylation^34^.

The detection of the early apoptotic cells developed by the BOZ treatments confirms our initial results on the cell viability of both cell lines. Dose-dependent apoptotic effect of BOZ was observed in the melanoma cells, whereas dose-dependency could not be detected in the myeloma cells. In contrast, other experiments carried out in this field^9,35^, we could detect a cell cycle arresting effect of BOZ on myeloma cells in the S-phase, while our results showed a G_2_/M-phase arrest on melanoma.

The most important, clinically relevant antitumor effect of BOZ was based on the reversible inhibition of the chymotrypsin-like protease activity of the proteasome^6^. Our present findings seem to be consistent with this research as the anti-proliferative effect of BOZ was associated with the inhibition of chymotrypsin-like proteasome activity in both cells. Concentration course study proved that the IC_50_ values of the proteasome inhibitory activity (A2058—IC_50_: 4.39 nM; U266—IC_50_: 1.49 nM) follow a similar tendency to the dose dependence of toxicity (A2058—IC_50_: 158 nM; U266—IC_50_: 2.17 nM).

Our present paper focuses on the drugs used for the treatment of BIPN, one of the most common side-effects of BOZ^36,37^. The neuroprotective antioxidant vitamins are capable of decreasing the symptoms of this side-effect, but due to their antioxidant property, they may also alter the antitumor effect of BOZ^17–20^. Despite the interest in cancer supportive care, no one—to the best of our knowledge—has studied how ALA and vit B1 are able to affect the antitumor activity of BOZ. We demonstrated the antagonizing effect of 100 μg/mL ALA on the anti-proliferative effect of 20 ng/mL BOZ in melanoma cells. The anti-proliferation-antagonizing effect could not evolve on myeloma cells. Surprisingly, no differences were found in the activation of p53 after treatment with the combinations of BOZ and ALA, or vit B1. In our investigations, we found that 100 μg/mL ALA can counteract the anti-proteasome activity of 20 ng/mL BOZ; however, alone it had no significant increasing effect on proteasome activity. Unfortunately, in case of the effects on cell apoptosis and on the cell cycle, the altered effect of co-treatments was not as dramatic as those revealed by the cell viability results. Contrary to expectations, the number of the early apoptotic cells was not decreased by 20 ng/mL BOZ + 100 μg/mL ALA and we did not find a significant difference between the sub G1 phase of the 20 ng/mL BOZ-treated and the 20 ng/mL BOZ + 100 μg/mL ALA-treated cells. This implies, that apoptosis and cell cycle are very complex cell biological processes, that are regulated at multiple points by several proteins, and this present study has only investigated a little part of these processes.

The increased ROS production was proved by a cell-based assay and by an imaging technique as an intrinsic characteristic of the melanoma cells, and it seems to be consistent with other research describing the role of oxidative stress in cancerous cells^38^. The ROS inducing effect of BOZ could be only detected by microscopy, not by the cell-based assay in melanoma cells, although, in myeloma cells, the cell-based assay was able to confirm the ROS promoting effect of BOZ. The antagonizing effect of 100 μg/mL ALA on the oxidative stress level found of melanoma cells could not be proved by Celldiscoverer 7. These rather contradictory results may be due to the difference in the specificity and sensitivity of the used assays and the high basal ROS content of the melanoma cells.

In this study we could demonstrate, that treatment with BOZ led to Caspase-3 and Heme Oxygenase-1 (HO-1) activation in both cell lines, and could upregulate the expression of Claspin only in melanoma cells. Caspases are characteristic in both intrinsic and extrinsic programmed cell death (apoptosis) and they are responsible for DNase activity^39^. Our data are consistent with previous results obtained on the cleavage of Caspase-3 post-BOZ treatment^40^. On the contrary, HO-1 is a bifunctional protein, it can trigger anti- and pro-oxidative alterations in cells^41^. HO-1 has been already investigated in multiple myeloma cells and our results correlated well with the data of Barrera et al., where HO-1 induction was associated with possible BOZ-resistance pathways^42^. In U266 cells, the upregulation of heat shock proteins, like HSP60 and HSP70 were also detectable, that is in good agreement with Shah et al.^43^. Claspin is a cytoprotective molecule, aiming to prevent the neoplastic transformation of cells as a tumor suppressor protein^44^. Therefore, Claspin can be overexpressed upon DNA damage and can promote S and G2 phase arrest that was reported in A2058 cells, but not in U266 cells after BOZ treatment in the present work.

In melanoma cells, it was confirmed that there is a decrease of the activation of these 3 upregulated proteins (cleaved-Caspase-3, Claspin and HO-1) after the cells were exposed to BOZ + ALA combination compared to the BOZ-treated cells. Similar effects on cleaved-Caspase-3, Claspin or HO-1 could not evolve in U266 cells. The absence of the significant difference on the relative level of these 3 apoptosis-related proteins between the BOZ-treated and the co-treated U266 cells have further strengthened our hypothesis, that the decrease in their activities may be in the background of the loss of the antitumor activity of BOZ found in A2058 cells.

This work calls the attention that BOZ may alter different pathways in the investigated melanoma and myeloma cell lines in vitro. Further data collection is required to unfold the dose-dependent correlation, how ALA affects the antitumor effect of BOZ. To sum up, the fact that 100 μg/mL ALA was able to disrupt the antineoplastic effect (the anti-proliferative effect, the proteasome inhibition effect and the expression of apoptosis-related proteins) of 20 ng/mL BOZ in melanoma cells in vitro, should point towards the proper use of cancer supportive care that must be in accordance with evidence-based medicine and must be under medical control.

## Materials and methods

### Materials

The tested concentrations of BOZ and the antioxidant vitamins (ALA and vit B1) were determined based on their maximal plasma concentrations that occur after the clinical use^23–25^. In each case, the dilutions were prepared right before the experiments due to the photosensitivity of compounds. BOZ (Velcade 3.5 mg; Janssen-Cilag GmBH, Neuss, Germany) was used at final concentrations of 20, 100 and 300 ng/mL, ALA (Thiogamma 600 Injekt; Wörwag Pharma GmbH & Co.KG, Stuttgart, Germany) at final concentrations of 10 and 100 µg/mL and vit B1 (Vitamin B1 50 mg Injection; Zentiva, Prague, Czech Republic) at final concentrations of 150 and 300 nmol/L^23–25^. All the assays were performed to examine the effects of BOZ, alone and combined with ALA or vit B1. In every case, untreated cells were utilized as controls for treated cells.

### Cell culturing

The effects of BOZ, as well as its combinations with ALA or vit B1, were investigated in two different types of cells: human myeloma (U266) and melanoma (A2058) cell lines which were obtained from European Collection of Authenticated Cell Cultures (ECACC, Salisbury, UK).

U266 myeloma cell line is growing in suspension (85051003 ECACC), while A2058 melanoma cell line is an adherent culture (91100402 ECACC). Both cell lines were grown in RPMI 1640 medium (Sigma Ltd. St. Louis, MO, USA), supplemented with 1% penicillin/streptomycin (Invitrogen Corporation, New York, NY, USA), 1% glutamine (Invitrogen Corporation, New York, NY, USA) and 10% fetal bovine serum (Invitrogen Corporation, New York, NY, USA). The medium of suspension U266 was removed in every 2nd-3rd day and fresh medium was supplied. In case of the adherent cells, for the subcultivation of the cells were washed with PBS (0.05 M phosphate buffer saline, pH = 7.4), then they were resuspended by enzymatic dissociation with 0.25% Trypsin–EDTA (Sigma Ltd. St. Louis, MO, USA) and split 1:3 to 1:6.

### Viability assay

In case of the adherent melanoma cell line, to evaluate the anti-proliferative effect of BOZ and its combinations with antioxidants, an innovative technique, impedimetry (xCELLigence SP; ACEA Biosciences, San Diego, CA, USA) was used. It provides a non-invasive, impedance-measurement method to get real-time information about the anti-proliferative effect of different compounds. Before treating and monitoring the cells, they were plated in a special 96-well plate (E-Plate 96 PET; ACEA Biosciences, San Diego, CA, USA) containing gold microelectrodes, which can monitor the electron flow in an electrically conductive solution, i.e. cell culture media. In the presence of the intact cells, the electron-flow is impeded by the continuous surface membrane of the cells thus the measured impedance is increased. This relates to the number, size, and morphology of the attached cells. The anti-proliferative effect of a compound can be tracked by the decrease of the impedance in parallel the decreasing number of viable cells. The data management can be performed by the RTCA 2.0 software (Real Time Cell Analyzer; ACEA Biosciences, San Diego, CA, USA) that can convert the measured impedance data to a unitless Cell Index (Cell Index = (Z_ti_ − Z_t0_)/F_i_; Z_ti_: impedance at individual time point; Z_t0_: impedance at start of experiment; F_i_: constant). Prior to the treatment, the baseline of the pure media and then for twenty-four hours the attachment and growth of non-treated cells (10^4^ cells/well) were registered in each experiment. Then the cells were treated with BOZ and the combinations of ALA and vit B1. Due to the suspension culture property of U266, we used the CellTiter-Glo Luminescent Cell Viability Assay (Promega, Madison, WI, USA). The cells (10^5^ cells/mL) were seeded in a white-walled 96-well plate. After an overnight culturing, the cells were treated with BOZ and the combinations of ALA and vit B1. After 24 h incubation, the CellTiter-Glo Reagent was added to the wells. Then the luminescence signal generated upon the ATP content of the cells was recorded by the Fluoroskan FL Microplate Fluorometer and Luminometer (Thermo Scientific, Waltham, MA USA). In similar experimental layouts, we were also able to determine the IC_50_ values (drug concentrations of 50% growth inhibition) for BOZ with xCELLigence Sp on A2058 cells in 10^–12^ − 10^–4^ M concentration range and with CellTiter-Glo on U266 cells in 10^–9^ − 10^–6^ M concentration range.

### Detection of p53 activation

To evaluate whether the examined compounds activate the p53 in the cells, we investigated the level of the phospho-p53 (S15). This is an intracellular staining method. The cells (5 × 10^5^ cells/0.5 mL) were fixed by 4% paraformaldehyde containing PBS and incubated for 10 min at room temperature. After a centrifugation step and harvesting the cells, the samples were in SAP/Triton buffer (0.1% saponin, 0.3% Triton-X, 0.05% NaN_3_ in Hanks’ Balanced Salt Solution) to permeabilize the plasma membrane. Following a centrifugation step, the supernatant was decanted, the cells were resuspended in the residual and 10 μl of the antibody conjugate (phycoerythrin-conjugated anti-human Phospho-p53 monoclonal antibody, R&D Systems, Minneapolis, MN, USA) was added to each sample. Prior to the analysis, the staining procedure needed 30–45 min incubation time at room temperature in the dark and following a centrifugation step the cells were resuspended in 200 μl PBS. The measurement was done by flow cytometry (BD FACSCalibur, Becton–Dickinson, San Jose, CA, USA).

### Apoptosis assay

In order to analyze the apoptotic effect of the compounds, we used a traditional technique, annexin V-assay, based on the translocation of the plasma membrane phosphatidylserine. The cells (7 × 10^4^ cells/mL) were seeded in a 24-well plate. Then the cells were treated with BOZ and the combinations of ALA and vit B. After the incubation time, the cells were harvested. In case of the adherent A2058 cells, the cells were resuspended with TrypLE reagent (Thermo Fisher Scientific, Waltham, MA, USA). The cells were then centrifuged and resuspended in 300 μL Annexin V Binding Buffer (Sony Biotechnology, Weybridge, UK). Then the cells were labeled with the fluorescein isothiocyanate (FITC) conjugated Annexin V (Ax V-FITC, Sony Biotechnology, Weybridge, UK) in the dark at room temperature. After the 15 min incubation of the staining procedure, the Ax V positive early apoptotic population can be measured by flow cytometry (BD FACSCalibur, Becton–Dickinson, San Jose, CA, USA) using the FL1 channel. The data management was performed by the CellQuest Pro (Becton–Dickinson, San Jose, CA, USA) and Flowing 2.5.1 software (Turku Centre of Biotechnology, Turku, Finland).

### Cell cycle analysis of fixed cells

NucleoCounter NC-250 (ChemoMetec, Allerod, Denmark) is a dual-channel fluorescent microscope. With this apparatus several different types of measurements can be performed, e.g. vitality or cell cycle assay. The current technique is based on the application of a DNA-staining dye, DAPI (4′,6-diamidino-2-phenylindole) that binds stoichiometrically to the double-stranded DNA but the amount of the DAPI-RNA complex is negligible. After subcultivation, the cells were washed with PBS and centrifuged at 500 × *g* for 5 min. The protocol requires 1 × 10^6^ to 2 × 10^6^/mL cells in suspension. As a fixative, the ice-cold 70% ethanol was utilized for at least 2 h. After the incubation time, there was a centrifugation step and the ethanol was decanted. Prior to the staining step, the cells were resuspended in PBS and centrifuged. Then the cells were resuspended in Solution 3, which contains 1 µg/mL DAPI and 0.1% Triton X-100 in PBS. The stained samples were loaded into 8-chamber slides and measured by NucleoCounter NC-250. The DNA-content was calculated by the NucleoView NC-250 software (ChemoMetec, Allerod, Denmark).

### Detection of the proteasome activity

To detect the proteasome activity of the cells after BOZ and co-treatments, we used a cell-based technique, the Cell-Based Proteasome-Glo Assay (Promega, Madison, WI, USA). The potential effect of the co-treatments on the anti-chymotrypsin-like protease activity of BOZ was determined by the specific luminogenic proteasome substrate Suc-LLVY-aminoluciferin sequence. The cleavage of this sequence by the active proteasome leads to a luciferase reaction that is responsible for the luminescent signal. The cells were seeded in the final volume of 90 μl (A2058: 6,000 cells/well; U266: 10,000 cells/well) in a white-walled 96-well plate (Thermo Scientific, Waltham, MA USA). Then the cells were treated with BOZ, antioxidants and the combinations for 24 h. After the incubation, 100 μL of the luminogenic reagent was added. Prior to the luminescent signal detection by the Fluoroskan FL Microplate Fluorometer and Luminometer (Thermo Scientific, Waltham, MA USA), the contents of the plate must be mixed at 700 rpm using a plate shaker for 2 min. The value of the luminescent signal of the sample blank was subtracted from all wells before data analysis. The raw data was normalized to the control.

### Luminescence-based measurement of intracellular H_2_O_2_ levels

The H_2_O_2_ generation was measured by the ROS-Glo H_2_O_2_ cell-based assay (Promega, Madison, WI, USA) in order to investigate the effect of BOZ and its combination on intracellular H_2_O_2_ levels. In this technique, after induction of H_2_O_2_, the H_2_O_2_ substrate is converted to a Luciferin precursor. In the consecutive step, this precursor can be converted to Luciferin. The appearing light signal is proportional to the level of intracellular H_2_O_2_ generated by the treatments. Both cell lines were seeded in white-walled 96-well (Thermo Scientific, Waltham, MA USA) plates at a density of 10,000 cells per well in 70 μL medium. Then the cells were treated with 10 μL of the different combinations of BOZ and the antioxidants and were incubated for 24 h. After 18 h long incubation, the H_2_O_2_ substrate was added to all wells in 20 μL. In the end, 100 μL of the luciferin detection reagent supplemented with the signal enhancer solution and D-cysteine, was added to all wells and luminescence was measured after 20 min incubation with the Fluoroskan FL Microplate Fluorometer and Luminometer (Thermo Scientific, Waltham, MA USA).

### Microscopic detection of intracellular ROS levels

The oxidative stress, induced by BOZ and its combinations, can be detected by the CellROX Deep Red reagent (Thermo Scientific, Waltham, MA USA) in the adherent A2058 cells. Due to the suspension characteristics of the U266 myeloma cell line, this technique was not performed on myeloma cells. The A2058 cells were plated in 96-well black-walled plate (Greiner Bio One, Frickenhausen, Germany) at a concentration of 10^5^ cells/mL. After an overnight culturing, the cells were treated with BOZ and the co-treatments for 24 h. As negative control 1,000 μM N-Acetyl-L-cysteine (NAC), as positive control 150 μM tert-Butyl hydroperoxide (tBHP) was used. Following the incubation time, the cells were stained with the CellROX Deep Red fluorogenic probe at a final concentration of 5 μM. In a reduced state this reagent is non-fluorescent, but upon oxidation, by intracellular ROS (O_2_^−^, H_2_O_2_, OH^−^) it becomes strongly fluorescent and emits light at 665 nm. The produced signals are localized in the cytoplasm. Along with the CellROX Deep Red reagent, a nuclear counterstain, Hoechst 33342 (Thermo Scientific, Waltham, MA USA) was also applied at a final concentration of 0.5 μg/mL. The cells were incubated with the reagents for 30 min and then washed 2 times. At the final step, cells were preserved for further analysis with 3.7% formaldehyde. The double-stained, fixed samples were imaged by Celldiscoverer 7 system using 5 × Plan-Apochromat λ/0.35 NA objective with 2 × tube lens (Carl Zeiss AG, Jena, Germany). Each well was photographed in minimum 20 different Z-planes, then the pictures were deconvoluted in order to enhance the signal-to-noise ratio using ZEN Blue 2.6 software (Carl Zeiss AG, Jena, Germany). The intensity values and the number of nuclei were determined by ImageJ software (NIH, USA).

### Cell lysate preparation

Cells were seeded into T-25 Flasks (2 × 10^6^ cells/flask) and the following day the cells were treated. After treatment, U266 and A2058 cells were washed with PBS and then A2058 cells were subjected to TrypLE treatment for dissociation. After subcultivation, the cells were centrifuged at 1,000 × *g* for 5 min. Then the supernates were transferred into clean tubes and the cells were rinsed with PBS and extracted with Lysis Buffer 17 (provided in the Human Apoptosis Array Kit). In order to separate also the apoptotic bodies induced by the treatments, the supernatants were centrifuged at 2,500 × *g* for 15 min at 4 °C, two times. Then the apoptotic-body containing pellet was extracted with Lysis Buffer 17. Before protein quantification, the lysed samples were mixed into each other and incubated with the lysis buffer for 20 min. Total protein quantity was evaluated by the colorimetric BCA assay (Thermo Scientific, Waltham, MA USA). Protein extracts (225 μg) were used for apoptosis array analysis.

### Proteome profiler human apoptosis array kit

Analysis of 35 different apoptosis-related proteins was conducted in accordance with the manufacturer’s instructions. First, the arrays were incubated in 4-well multi-dish with a block buffer, Array Buffer 1. Then the buffer was removed and every array was incubated with the samples overnight at 4 °C on a rocking platform shaker. The unbound proteins were removed and the arrays were washed with 20 mL washing buffer three times. Detection Antibody Cocktail was added to all arrays for 1 h. The arrays were washed again with the washing buffer three times. A specific biotinylated detection antibody was added to all wells on a rocking platform shaker for 30 min. Then the spots were visualized by the Chemi Reagent Mix and evaluated by Bio-Rad Chemidoc XRS + system. The mean intensities of the spots were measured by Image Lab Software (BIO-RAD, USA).

### Statistical analysis

Evaluation of the results was performed by using the MS Excel and OriginPro 8.0 (OriginLab Corporation, Northampton, MA, USA) software. The data were statistically analyzed by the use of one-way analysis of variance (ANOVA) followed by Fishers Least Significant Difference (Fishers LSD) post hoc test. Every treated sample was compared to the matching untreated, medium control. The result of this analysis is found directly above the columns. If there is any statistical difference between the means of the group members, the level of significance is presented on a horizontal line connecting the two significantly different data. The image quantification was performed by ImageJ software. The relative protein levels were determined by Image Lab software. The levels of significance are shown as follows: x: P < 0.05; y: P < 0.01; z: P < 0.001. The combined effects of different concentrations of BOZ with ALA were determined, and the combination index (CI) values were evaluated using CompuSyn software. Combination index < 1, = 1, or > 1 represents synergism, additive effect or antagonism, respectively.

## Supplementary information

Supplementary information.
