# Supplementary material for: Alpha-lipoic acid alters the antitumor effect of bortezomib in melanoma cells in vitro
Source: Sci Rep. 2020 Aug 31;10:14287. doi: 10.1038/s41598-020-71138-z (PMC7459291; doi:10.1038/s41598-020-71138-z)
Supplement: Supplementary file 1 — Supplementary information. [file 41598_2020_71138_MOESM1_ESM.docx]

## TITLE PAGE

**Article title: Alpha-lipoic acid alters the antitumor effect of bortezomib in melanoma cells in vitro**

**Authors: Angéla Takács^1^, Eszter Lajkó^1^, Orsolya Láng^1^, Ildikó Istenes^2^, László Kőhidai^1*^**

**Affiliations:**

^1^Department of Genetics, Cell- and Immunobiology, Semmelweis University, Budapest, Hungary

^2^1^st^ Department of Internal Medicine, Semmelweis University, Budapest, Hungary.

*** Corresponding author**:

Email address: kohlasz2@gmail.com


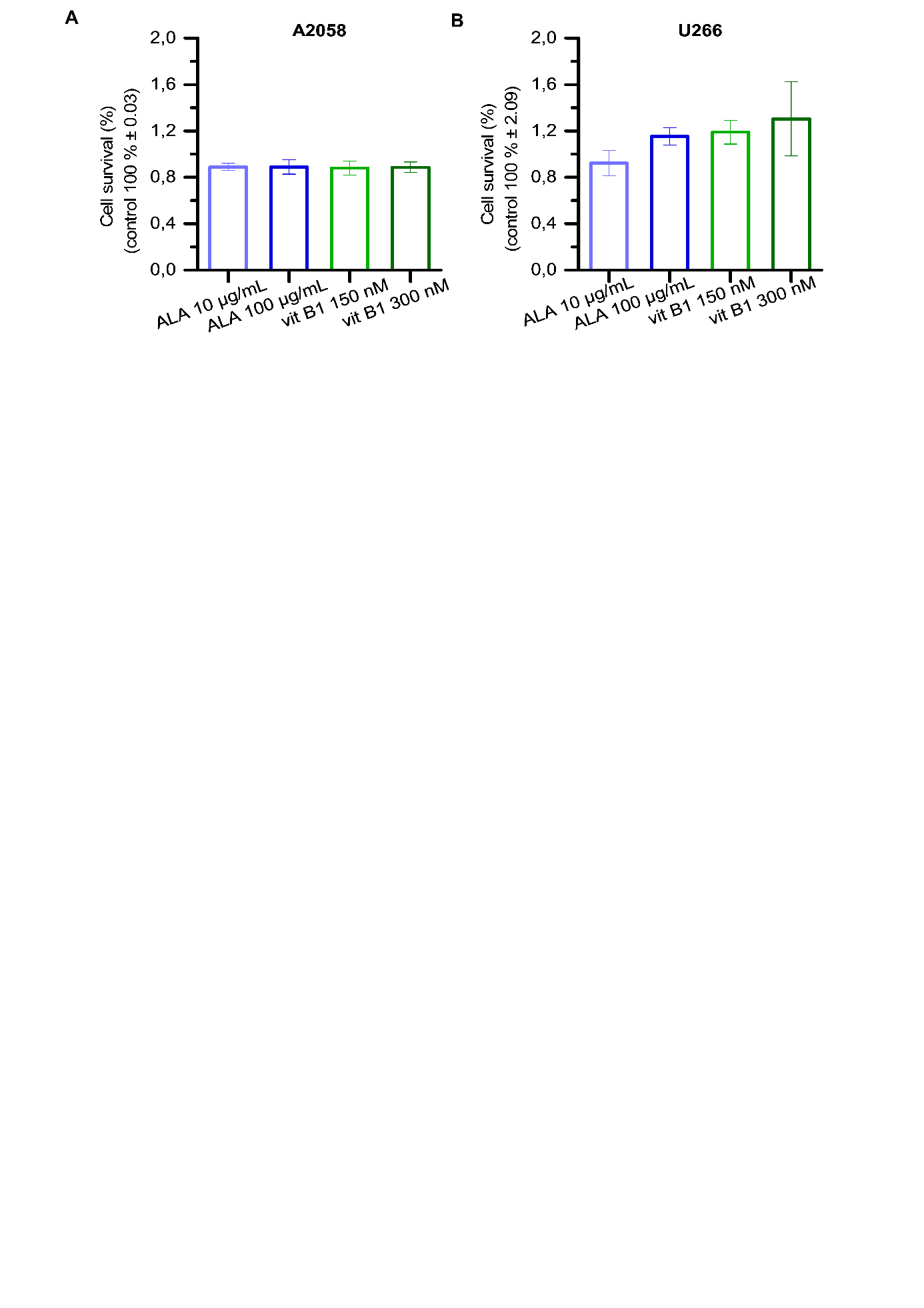


**Supplementary Figure S1:** Influence of alpha-lipoic acid and vitamin B1 on A2058 (**A**) and U266 cells (**B**) after 24 h incubation. Cells were treated with therapeutic agents as indicated. Data are given as mean values ± standard deviation (SD), (n=3). The levels of significance are shown as follows: x: P < 0.05; y: P < 0.01; z: P < 0.001, determined by the One-way ANOVA test followed by Fishers LSD *post hoc* test.

**Supplementary Table S1:** Analysis of the level of the activated p53 of A2058 and of U266 cells after 24h incubation with bortezomib (BOZ), alpha-lipoic acid (ALA) and Vitamin B1 (vit B1) as indicated. The ratio of mean fluorescence intensity is reported. The table shows representative data of 2 identical experiments.

|  | A2058 (RFI) | U266  (RFI) |
| --- | --- | --- |
| BOZ 20 ng/mL | 1.54 | 1.21 |
| + ALA 10 μg/mL | 1.56 | 1.17 |
| + ALA 100 μg/mL | 1.50 | 1.16 |
| + vit B1 150 nM | 1.53 | 1.17 |
| + vit B1 300 nM | 1.54 | 1.13 |
| BOZ 100 ng/mL | 1.47 | 1.22 |
| + ALA 10 μg/mL | 1.53 | 1.15 |
| + ALA 100 μg/mL | 1.58 | 1.16 |
| + vit B1 150 nM | 1.72 | 1.19 |
| + vit B1 300 nM | 1.68 | 1.22 |
| BOZ 300 ng/mL | 1.73 | 1.21 |
| + ALA 10 μg/mL | 1.66 | 1.15 |
| + ALA 100 μg/mL | 1.60 | 1.14 |
| + vit B1 150 nM | 1.66 | 1.05 |
| + vit B1 300 nM | 1.76 | 1.05 |
| ALA 10 μg/mL | 1.078 | 1.03 |
| ALA 100 μg/mL | 1.10 | 0.98 |
| vit B1 150 nM | 0.92 | 0.95 |
| vit B1 300 nM | 1.10 | 0.93 |
| **Table 1. compares the RFI data of the two different cell lines, A2058 and U266 after incubating with BOZ and the indicated combinations for 24h.** The ratio of mean fluorescence intensity (RFI) was calculated by normalising the mean fluorescence intensity of the treated cells to the control cells. The figure shows representative data of 2 identical experiments. |  |  |

RFI: the ratio of the mean fluorescence intensity; MFI: mean fluorescence intensity; RFI = treated cells p53 MFI/control cells p53 MFI.


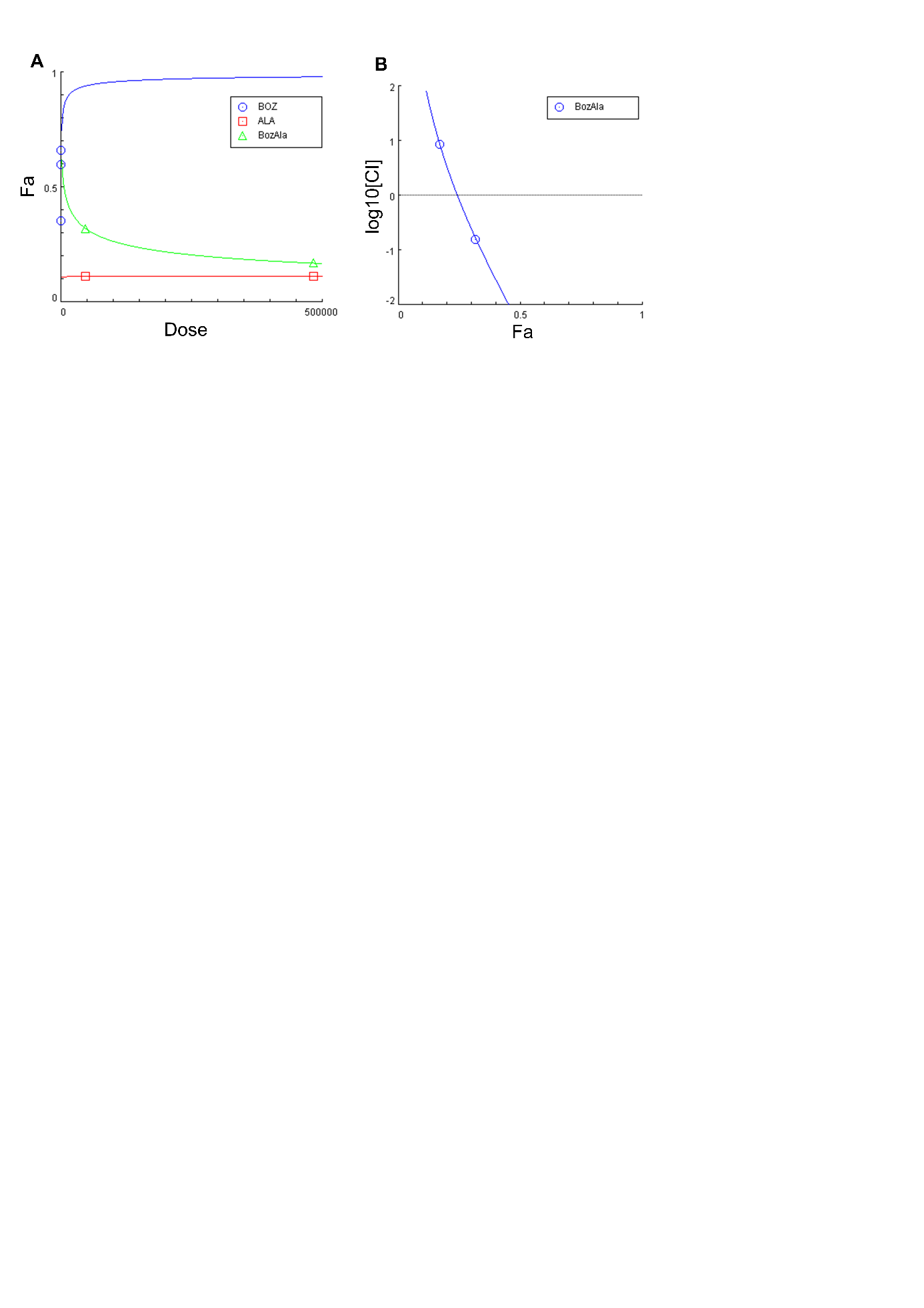


**Supplementary Figure S2:** Concentration-response effects on A2058 cells following 24 h of treatment with BOZ (bortezomib), ALA (alpha-lipoic acid) and their combination (**A**) depicted by CompuSyn Software. Logarithmic combination index plot (**B**) for BOZ (20 ng/mL) + ALA (10-100 μg/mL) co-treatments are plotted. Data obtained via CompuSyn analysis. CI: combination index; Fa: fraction affected.


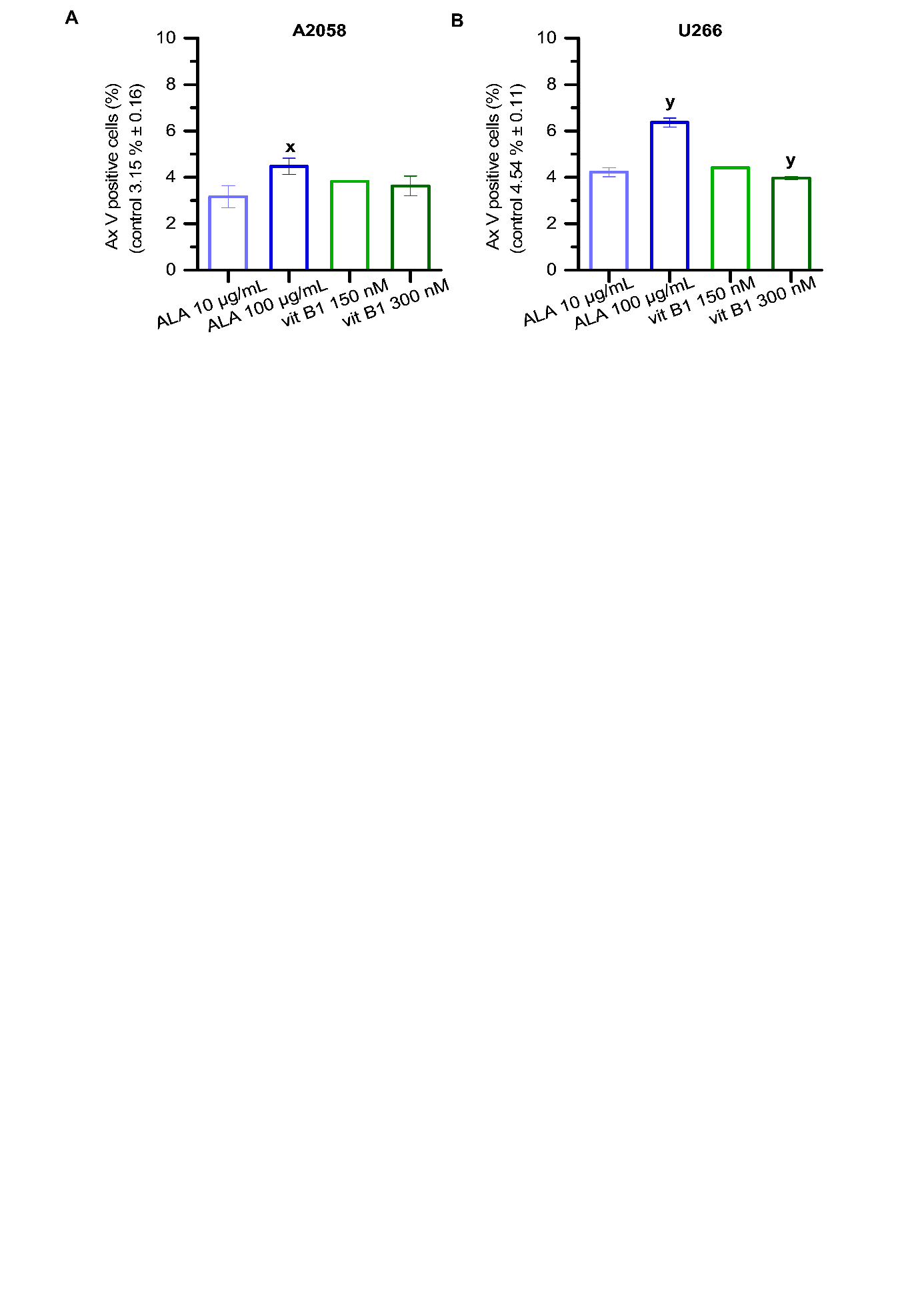


**Supplementary Figure S3:** Apoptosis in A2058 (**A**) and U266 (**B**) cells after 24 h long incubation with 10 or 100 μg/mL alpha-lipoic acid (ALA) and 150 or 300 nM vitamin B1 (vit B1) analyzed by Annexin V assay. Data are given as mean values ± standard deviation (SD) (n=2). The levels of significance are shown as follows: x: P < 0.05; y: P < 0.01; z: P < 0.001, determined by the One-way ANOVA test followed by Fishers LSD *post hoc* test.


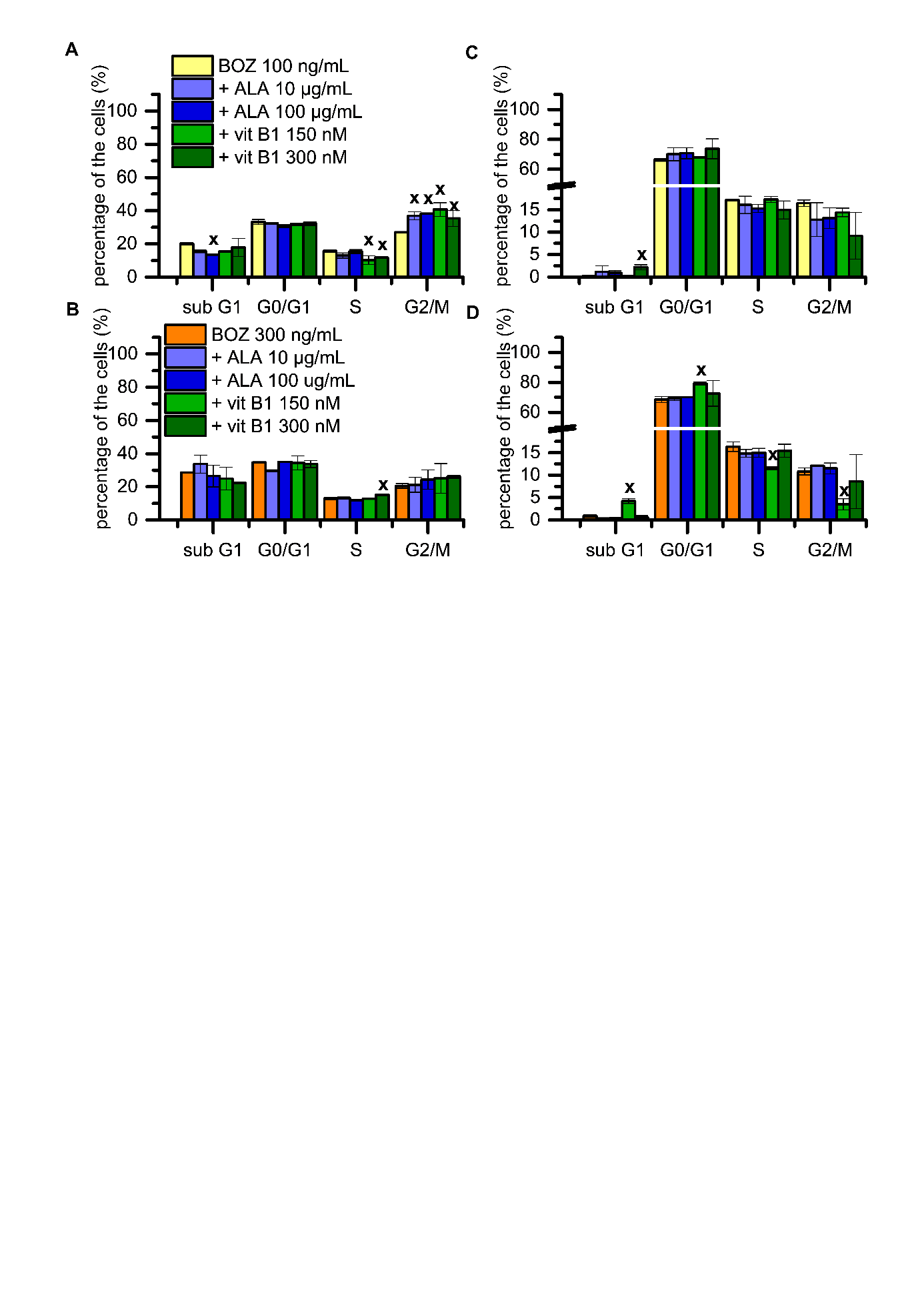


**Supplementary Figure S4**: Cell cycle analysis of A2058 (A-B) and U266 (C-D) cell lines after 24 h long incubation with 100 and 300 ng/mL bortezomib (BOZ) and combinations of 20 ng/mL BOZ + 10 or 100 μg/mL alpha-lipoic acid (ALA) and 150 or 300 nM vitamin B1 (vit B1) analyzed by NucleoCounter^TM^. Data are given as mean values ± standard deviation (SD) (n=2). The levels of significance are shown as follows: x: P < 0.05; y: P < 0.01; z: P < 0.001, determined by the One-way ANOVA test followed by Fishers LSD post hoc test.


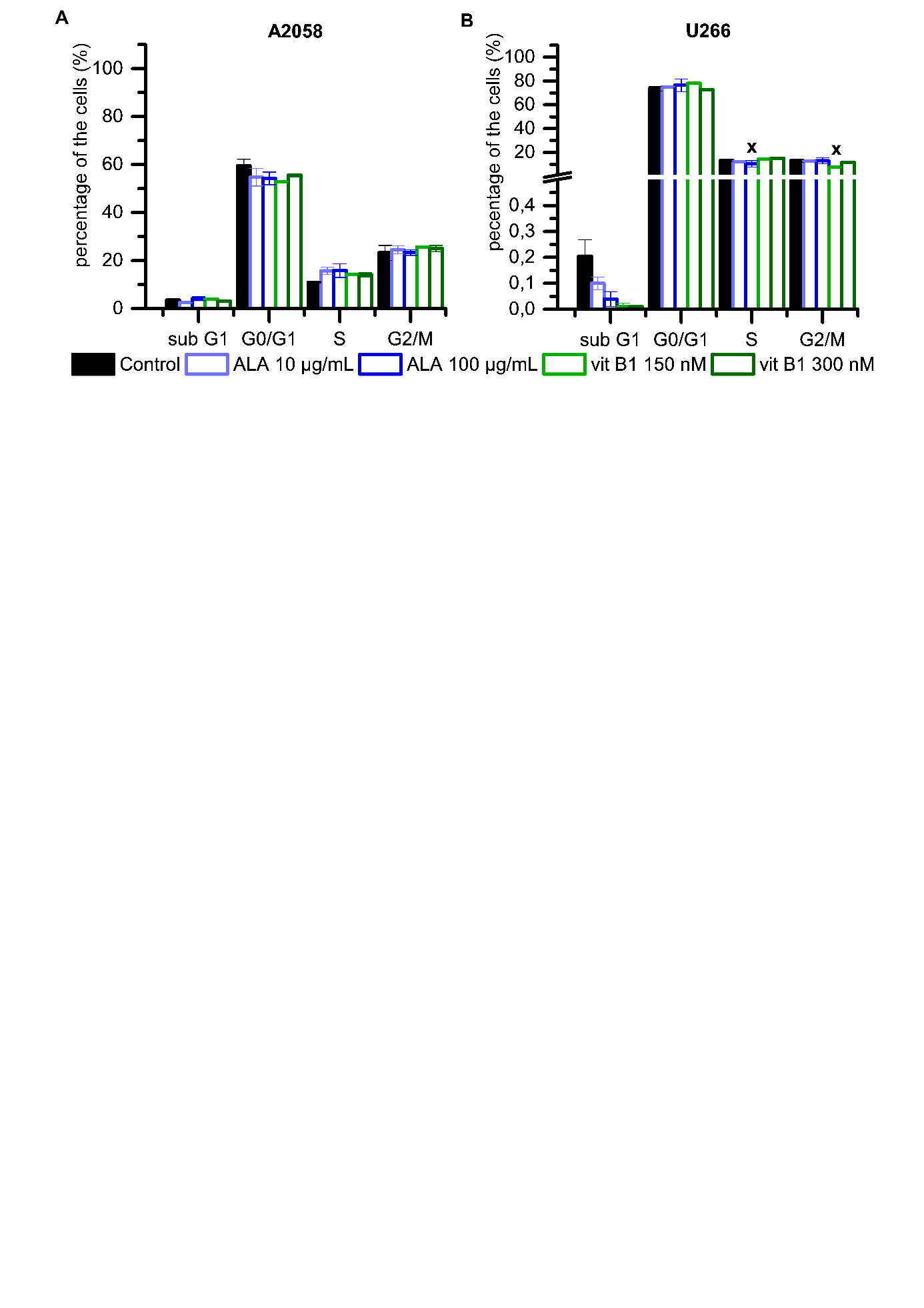


**Supplementary Figure S5:** Cell cycle analysis of A2058 (**A**) and U266 (**B**) cell lines after 24 h long incubation with 10 or 100 μg/mL alpha-lipoic acid (ALA) and 150 or 300 nM vitamin B1 (vit B1) analyzed by NucleoCounter^TM^. Data are given as mean values ± standard deviation (SD) (n=2). The levels of significance are shown as follows: x: P < 0.05; y: P < 0.01; z: P < 0.001, determined by the One-way ANOVA test followed by Fishers LSD *post hoc* test.


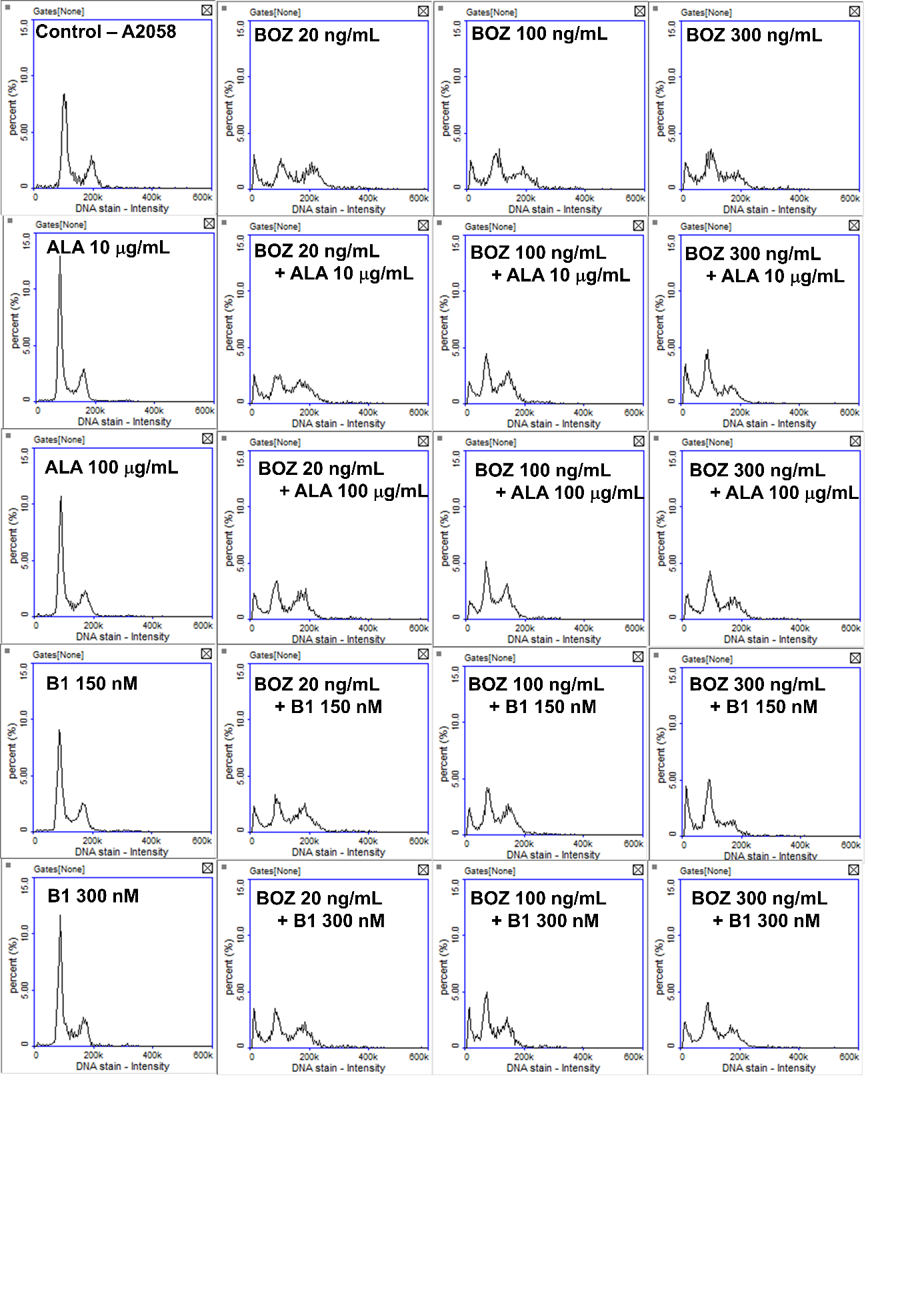


**Supplementary Figure S6:** The raw data of the cell cycle analysis of A2058 cell. The cells were treated as indicated and then were analyzed by NucleoCounter^TM^.


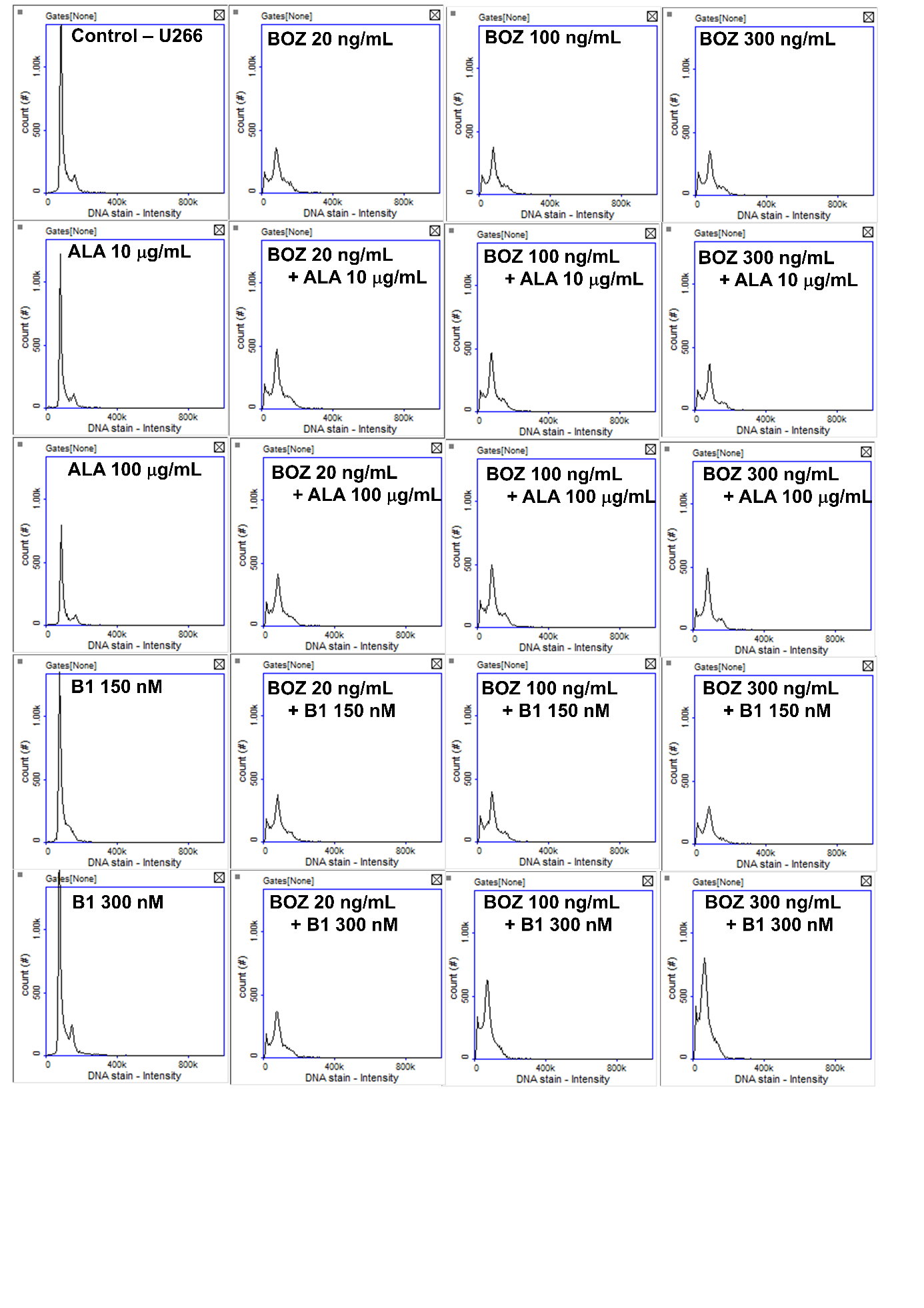


**Supplementary Figure S7:** The raw data of the cell cycle analysis of A2058 cell. The cells were treated as indicated and then were analyzed by NucleoCounter^TM^.


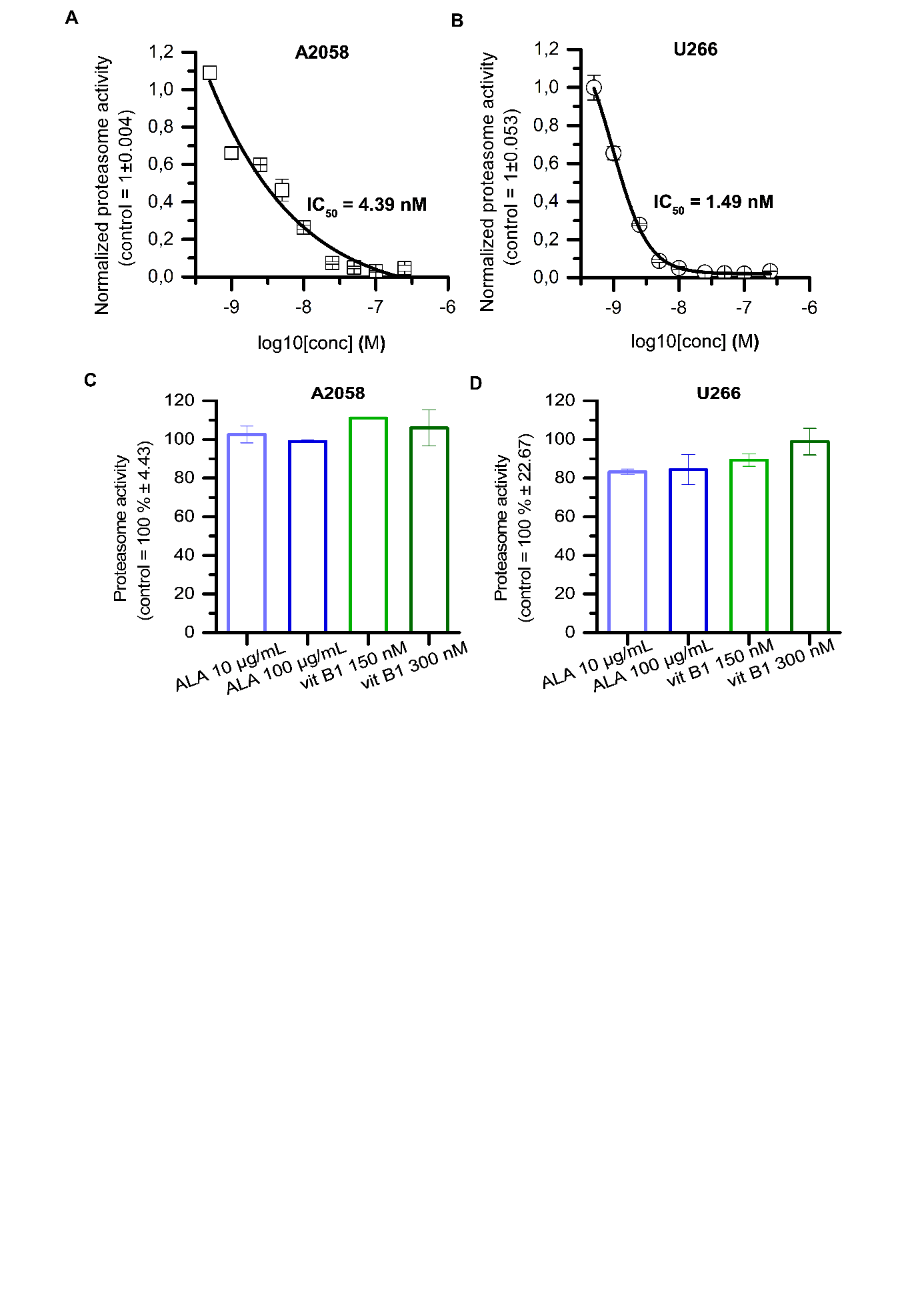


**Supplementary Figure S8:** Dose-response curves of BOZ in A2058 (**A**) and U266 (**B**) cells. Cells were treated with BOZ (concentration range: 10^-9^-10^-6.5^ M) for 24 h. The data are normalized to the control wells. The IC_50_ value of BOZ was determined by fitting a sigmoidal dose-response curve to the data, using Origin Pro 8.0. The effect of 10 or 100 μg/mL alpha-lipoic acid (ALA) and 150 or 300 nM vitamin B1 (vit B1) on the chymotrypsin-like activity of the proteasome in A2058 (**C**) and in U266 (**D**) cells. Cells were treated with therapeutic agents as indicated for 24 h. The data were normalized to the control wells. Data are given as mean values ± standard deviation (SD), (n=3). The levels of significance are shown as follows: x: P < 0.05; y: P < 0.01; z: P < 0.001, determined by the One-way ANOVA test followed by Fishers LSD *post hoc* test.


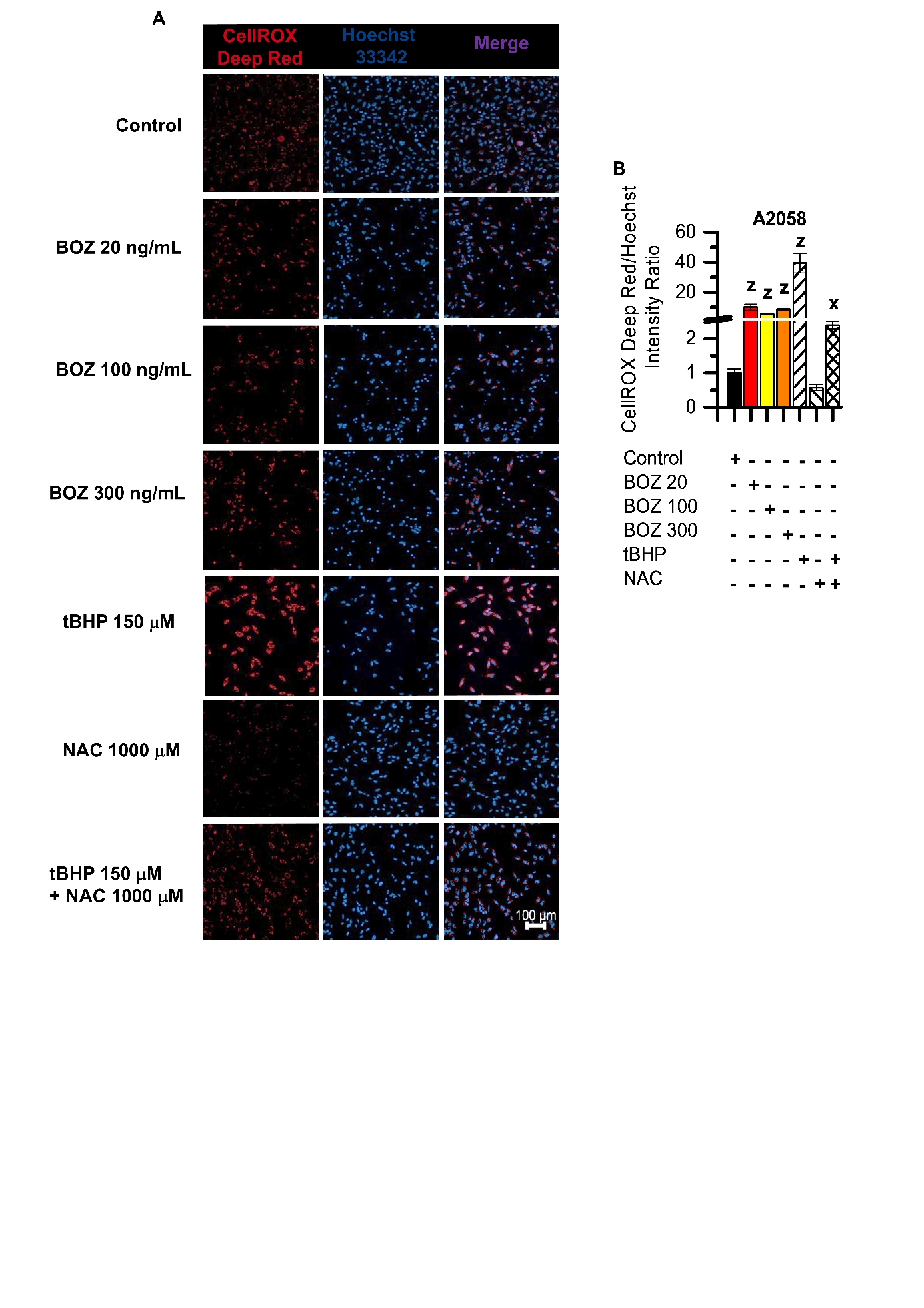


**Supplementary Figure S9:** (**A**) Melanoma cells were treated as indicated for 24 hours. The cells were imaged on Zeiss Celldiscoverer 7 using 10x magnification. The scale bar represents 100 μm. (**B**) Comparison of the intensity values of the red channel normalized to the cell nuclei. The data were analyzed using ImageJ software. Data in duplicates were expressed as mean ± standard deviation. The levels of significance are shown as follows: x: P < 0.05; y: P < 0.01; z: P < 0.001, determined by the One-way ANOVA test followed by Fishers LSD *post hoc* test.

**
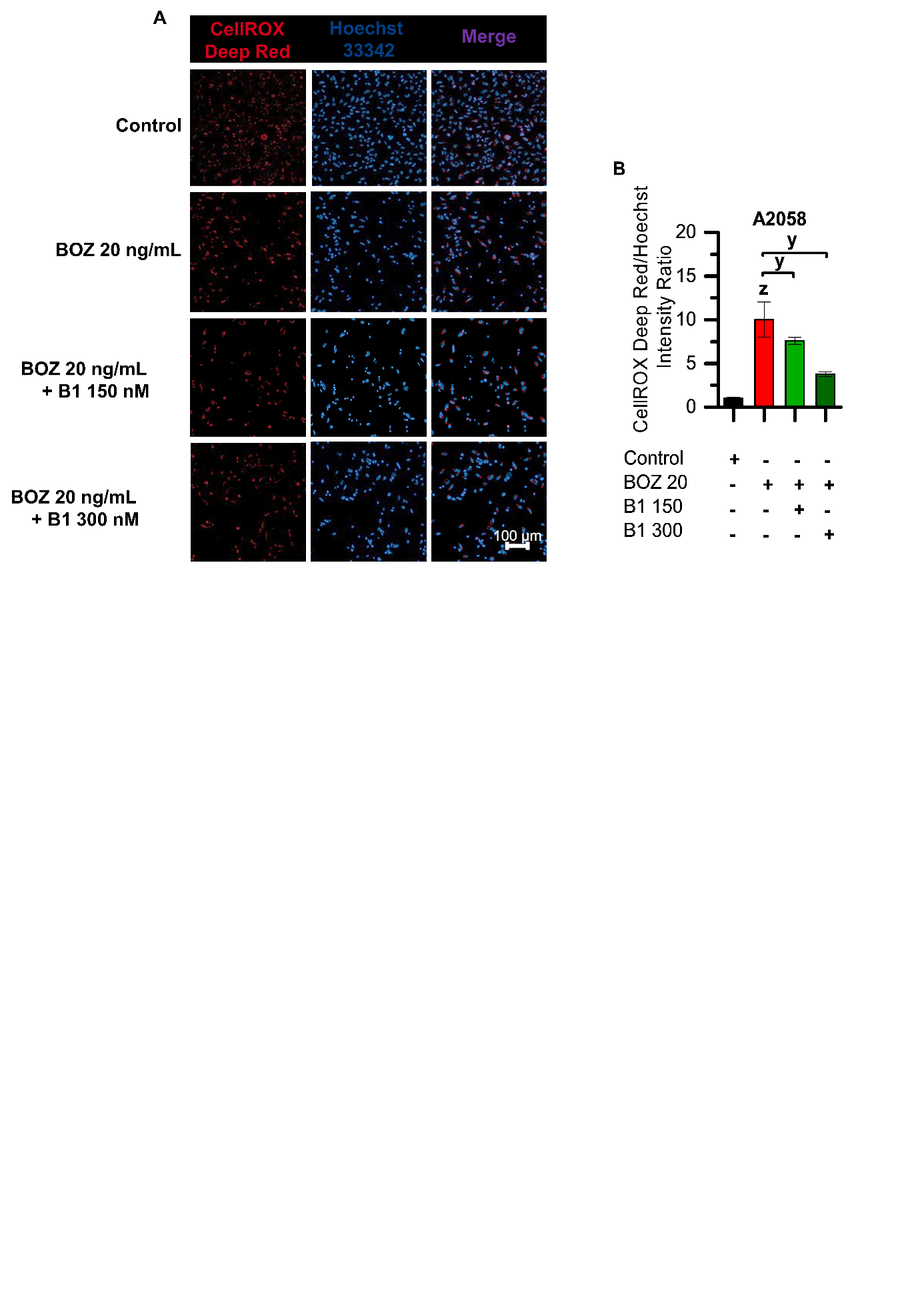
Supplementary Figure S10:** (**A**) Melanoma cells were treated as indicated for 24 hours. The cells were imaged on Zeiss Celldiscoverer 7 using 10x magnification. The scale bar represents 100 μm. (**B**) Comparison of the intensity values of the red channel normalized to the cell nuclei. The data were analyzed using ImageJ software. Data in duplicates were expressed as mean ± standard deviation. The levels of significance are shown as follows: x: P < 0.05; y: P < 0.01; z: P < 0.001, determined by the One-way ANOVA test followed by Fishers LSD *post hoc* test.


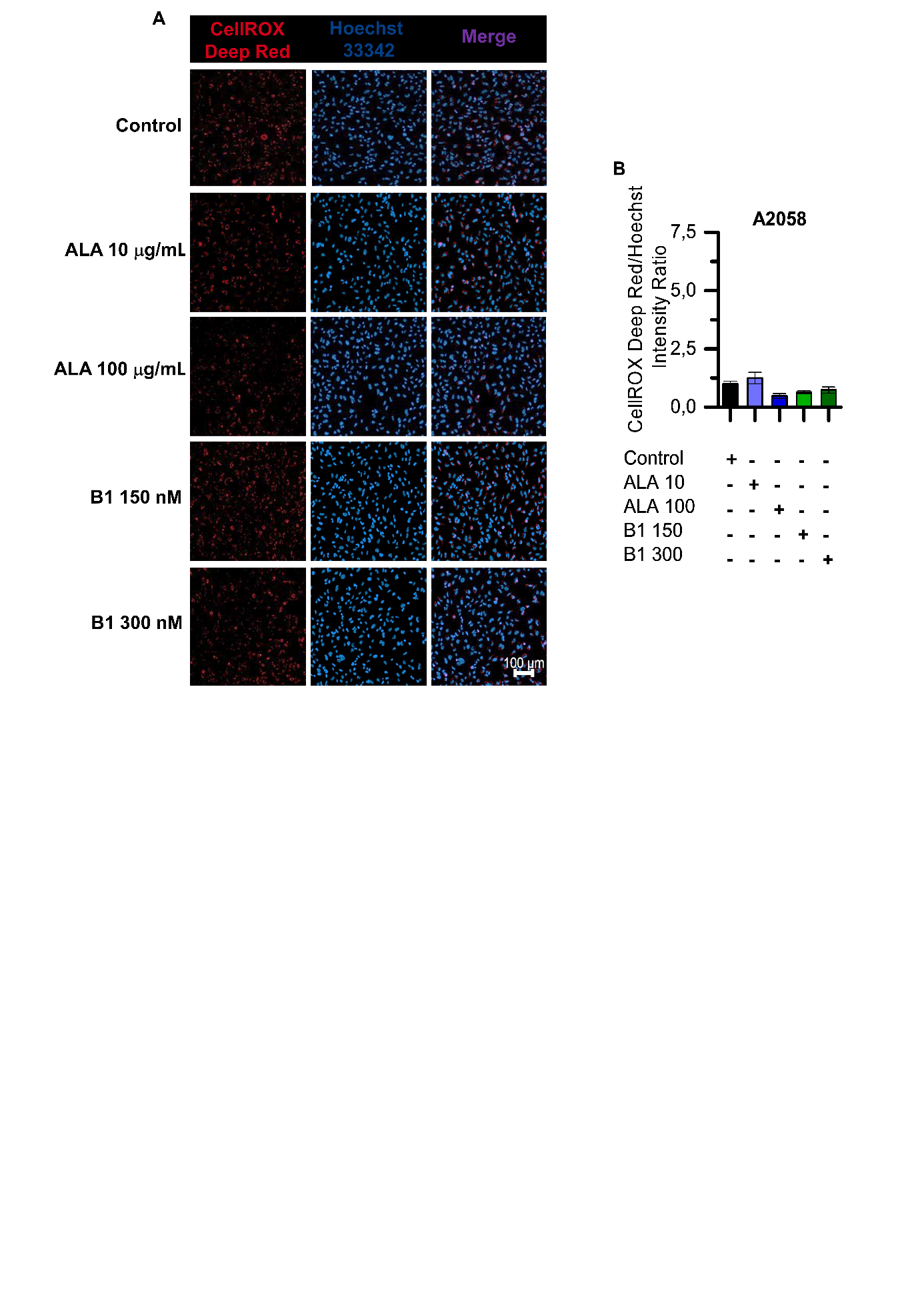


**Supplementary Figure S11:** (**A**) Melanoma cells were treated as indicated for 24 hours. The cells were imaged on Zeiss Celldiscoverer 7 using 10x magnification. The scale bar represents 100 μm. (**B**) Comparison of the intensity values of the red channel normalized to the cell nuclei. The data were analyzed using ImageJ software. Data in duplicates were expressed as mean ± standard deviation. The levels of significance are shown as follows: x: P < 0.05; y: P < 0.01; z: P < 0.001, determined by the One-way ANOVA test followed by Fishers LSD *post hoc* test.


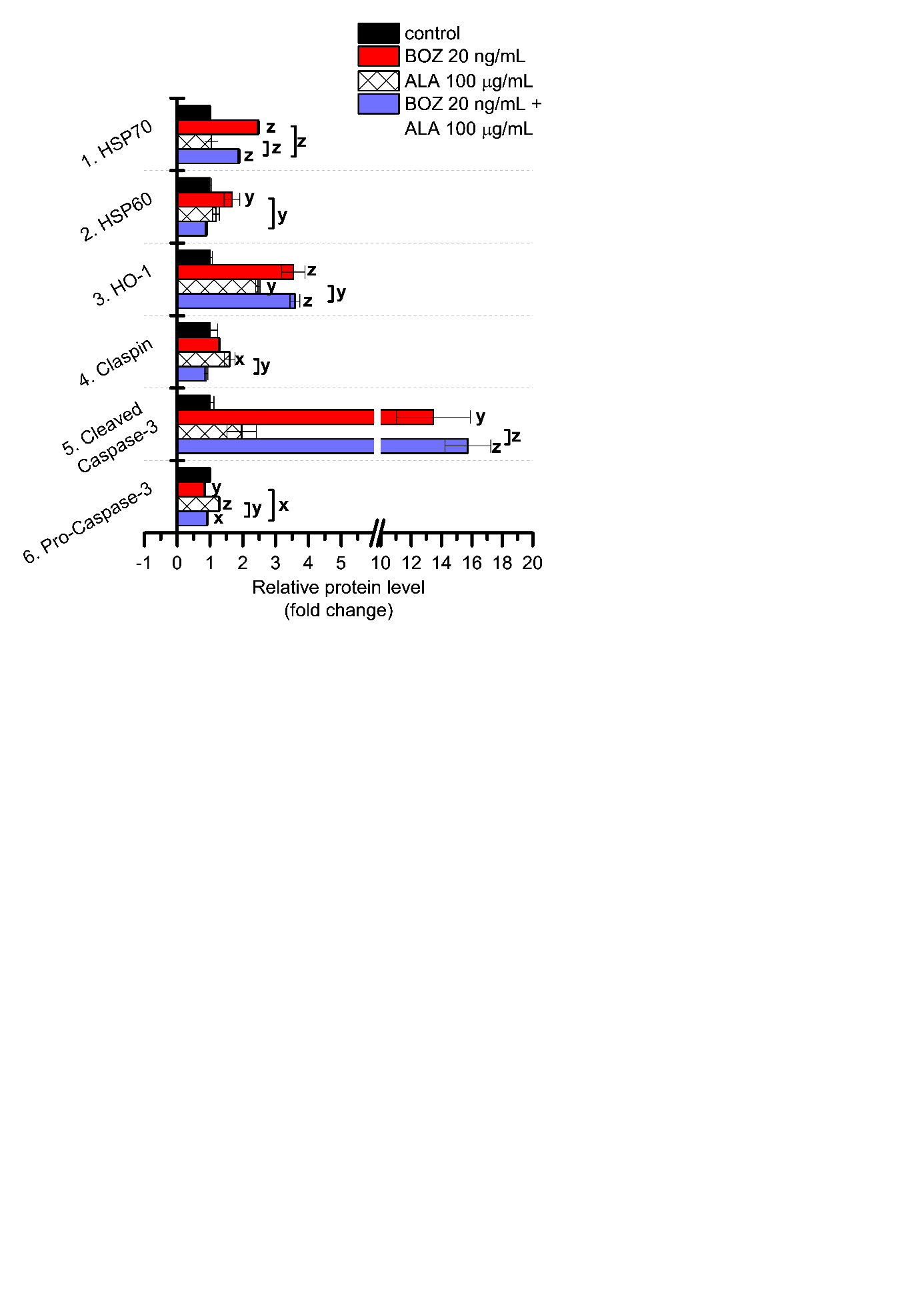


**Supplementary Figure S12:** Proteome profiling of U266 cells after treated with 20 ng/mL BOZ, 100 μg/mL ALA and their combination for 24 hours. Relative protein levels were analyzed using Image Lab 6.0.1 Software. The levels of significance are shown as follows: x: P < 0.05; y: P < 0.01; z: P < 0.001, determined by the One-way ANOVA test followed by Fishers LSD post hoc test.
